# Organizational behavior in international strategic alliances and the relation to performance – a literature review and avenues for future research

**DOI:** 10.1007/s11301-022-00268-7

**Published:** 2022-05-10

**Authors:** Marius G. Gehrisch, Stefan Süß

**Affiliations:** Lehrstuhl für BWL, insb. Arbeit, Personal und Organisation, Heinrich-Heine-Universität Düsseldorf, Universitätsstraße 1, 40225 Düsseldorf, Germany

**Keywords:** Organizational behavior, International strategic alliances, Performance, Literature review, D23, F53, M16

## Abstract

This paper presents a systematic literature review of the research on behavioral factors influencing the performance of international strategic alliances. After capturing the relevance of the research field, we observe the distribution of publications and derive quantitative metrics. Further, we focus on the terms related to alliance performance used in this research domain. Then, the results regarding the behavioral factors of influence on the individual, group-related and organizational level and their relation to alliance performance are stated. Our analysis ascertains that some factors are present on at least two behavioral levels and are understood differently on each level, leading to a certain multidimensionality. Therefore, we develop a categorization that cross all behavioral levels based on four broad categories: relational factors, learning and knowledge, conflict, and other (unrelated) factors. Based on this analysis, we identify avenues for future research. Beside methodological needs for research, gaps concerning the multidimensionality we recognized and various influencing factors are identified, as ambiguous results are apparent or other factors have been scarcely analyzed so far.

## Abstract (de)

Die vorliegende Studie umfasst eine systematische Analyse der Literatur zu verhaltenswissenschaftlichen Faktoren und deren Einfluss auf die Performance von internationalen strategischen Allianzen. Nach Erfassung der Relevanz des Forschungsfeldes, betrachten wir die Verteilung der in die Analyse einfließenden Artikel und leiten quantitative Metriken ab. Ferner wird der in diesem Forschungsfeld verwendeten Performance-Begriff von internationalen strategischen Allianzen untersucht. Anschließend wird der Zusammenhang zwischen den verhaltenswissenschaftlichen Einflussfaktoren, auf individueller, gruppenbezogener und organisationaler Ebene, und der Performance von internationalen strategischen Allianzen analysiert und die gewonnenen Ergebnisse dargelegt. Unsere Analyse stellt fest, dass einige Faktoren auf mindestens zwei Verhaltensebenen vorhanden sind und diese Faktoren auf manchen Ebenen unterschiedlich verstanden werden können, was zu einer gewissen Mehrdimensionalität führt. Daher entwickeln wir eine Kategorisierung, die sich über alle Verhaltensebenen erstreckt und auf vier Kategorien basiert: Beziehungsfaktoren, Lernen und Wissen, Konflikte und andere (nicht verwandte) Faktoren. Auf der Grundlage dieser Analyse zeigen wir Wege für die zukünftige Forschung auf. Neben methodischem Forschungsbedarf werden Lücken in Bezug auf die von uns erkannte Mehrdimensionalität identifiziert. Daneben heben wir verschiedene Einflussfaktoren hervor, deren Einflüsse auf oder Zusammenhänge mit anderen Faktoren uneineindeutig sind oder bisher kaum analysiert wurden.

## Introduction

For decades, *international strategic alliances (ISA)* have provided the opportunity for multinational enterprises to enter foreign markets (Haase and Franco 2015) and improve organizational learning (Parmigiani and Rivera-Santos 2011). Therefore, ISA became essential to enhance innovation and be competitive in a globalized world (Nielsen and Nielsen 2009; Schweitzer 2014). For example, during the COVID-19 pandemic, medical companies like BioNTech and Pfizer cooperated globally to develop and distribute vaccines (European Commission 2020; FAZ.net 2021). In a number of industries ISA have always been a prominent way for international operating companies to achieve competitive advantage (e.g., Business Insider 2021).

The literature describes ISA as international and voluntary inter-firm collaborations involving two or more legally distinct organizations that actively participate in decision-making and investment-activities over a given period of time, in order to attain mutually defined goals (e.g., co-development or sharing of products, technologies or services) (Gulati 1998; Nielsen and Gudergan 2012). However, the achievement of these set goals, often defined as *(alliance) performance* (Ariño 2003), has always been deficient (Madhok 2006; Lu and Ma 2015). To identify potential factors influencing alliance performance, especially the literature in the field of strategic and international management has dealt with this topic (e.g., Sim and Ali 2000; Chattopadhyay and Bhawsar 2016). But, ISA still regularly fail to achieve their set goals resp. determined performance (Farrell et al. 2011; Park et al. 2012; Nippa and Beechler 2013; Dadfar et al. 2014). Hence, recent research postulates to take additional research lines, in particular insights resulting from *organizational behavior*, into consideration (Nippa and Reuer 2019). Several studies have indicated that behavioral constructs, commonly categorized on the individual, group-related and organizational level (e.g., Schuler 2001; Schnake and Dumler 2003), can impact the performance of ISA (e.g., Wai On et al. 2013). Thus, practitioners, but particularly researchers are interested in understanding what behavioral constructs could influence alliance performance. Insights grounded in the literature of organizational behavior could help to minimize the research gap that is resulting from the still imperfect understanding of alliance performance.

The *research field that addresses the performance of ISA* arose when authors started discussing this topic in the 1970s (e.g., Peterson and Schwind 1977; Peterson and Shimada 1978). Some scientists claim methodological weaknesses (Robson et al. 2006a, b; Meier 2011), and others describe this research domain as fragmented (Isidor et al. 2012). Moreover, management research, the formation of ISA and economic surroundings have changed per se (Westney 2011; Williams 2011). In addition, various authors have pointed out that most research in this area tends to focus on strategic aspects. For example, many authors have analyzed the partnering companies’ equity distributions, control and decision-making processes (Roy Chowdhury 2009; Chattopadhyay and Bhawsar 2016; Iriyama et al. 2014; Parameswar and Dhir 2019), political environments (Zheng and Larimo 2014; Chang et al. 2015), market conditions (Reuer 2001), or potential resource complementarities (Dong et al. 2019) that impact alliance performance. Compared to studies that deal with such strategic aspects, articles that have addressed behavioral variables are still scarce. Nippa and Reuer (2019) explicitly suggested that an understanding concerning behavioral variables is important and should be focused on in upcoming studies. Though, research in this area is already increasing (Gomes et al. 2016), as trust and commitment between the partnering companies (Nisar et al. 2018; Owens et al. 2018), knowledge transfer/organizational learning (Park and Vertinsky 2016; Zhang et al. 2018) and national/organizational cultural differences have been analyzed (Mohr and Puck 2005; Feng et al. 2016; Pesch and Bouncken 2018; Low et al. 2020).

The field features some literature reviews, but most are broad, addressing factors like company size, strategic relatedness, cooperation, and international experience that could influence the performance of ISA (e.g., Christoffersen 2013; Gomes et al. 2016). However, only one review has addressed aspects of the behavioral side that are linked to ISA by concentrating on factors like trust, commitment, conflict reduction, and communication (Robson et al. 2006a, b). But, this review was published more than a decade ago. So, more recent empirical studies or constructs in the realm of organizational behavior (e.g., job satisfaction, stress) have not been recognized. In addition, some reviews that have had a behavioral approach (e.g., Meier 2011) have dealt with a single aspect (e.g., knowledge management) instead of mapping a comprehensive picture and have not included a broad range of behavioral constructs that could be helpful in understanding what influences the performance of ISA.

Hence, a *systematic, comprehensive literature review* that examines behavioral constructs resp. therein embedded influencing factors on the individual, group-related and organizational level and depicts the state of the research in this field is still lacking. We believe that it could be helpful to determine which levels and which related constructs have been dealt with more extensively and which levels should be focused on in more detail. By doing so, one can get a broad overview of the fragmented research findings and identify different gaps in research. Moreover, this review will contribute to the already existing body of ISA-related literature that is grounded in strategic and international management, as we apply a behavioral lens to understand alliance performance comprehensively. Besides, research regarding organizational behavior will benefit as well, since we add ISA as a special organizational form to behavioral research and, thus, consider a novel object of analysis by systematically analyzing the available articles that fit to our research approach.

Therefore, *this study* aims to systematically review the state of research regarding behavioral constructs in the context of ISA and their performance using three steps: firstly, a quantitative analysis of the number of relevant publications; secondly, a qualitative, content-based analysis of selected publications; and thirdly, a listing of implications for future research. Thus, the paper is structured as follows: First, the research questions that guide the review are derived, followed by the description of the conceptual background as well as the applied method and the thereby obtained literature sample. Then, the results from the review are presented. Next, the discussion and suggestions for future research are presented. Finally, the study ends with limitations and concluding remarks that contain some practical implications as well.

## Research questions

In a first step, we want to describe the current state of research concerning how alliance performance is influenced by behavioral constructs. We identify the number of articles published over time and ascertain the academic journals that published them. By doing this, we can show to what extent research has developed so far and describe the applied methodologies of the articles. This research approach allows to overview the fragmented research field and recognize time periods of high and low numbers of publications, so we propose the following research question regarding the composition of academic research on this topic:

*RQ1 What is the state of research, including its development over time, in terms of academic journals and methodologies?*

Next, we establish an overview of the term *alliance performance*, defined as the degree of accomplishment of partners’ goals (Ariño 2003). We regard alliance performance as a corporate indicator of success, which is broadly advocated by the literature (e.g., Mohr and Puck 2007; Yan et al. 2010; Baughn et al. 2011). So, we use no other performance-related term (e.g., individual-, innovation-, marketing-performance), as we focus on corporate outcomes.

Performance has always been a controversial topic (Venkatraman and Ramanujam 1986; Bener and Glaister 2010). Some authors depict alliance performance as an *objective* construct (Geringer and Hebert 1991; Damanpour et al. 2012; Gómez-Miranda et al. 2015) that comprises of several measurable factors – normally, financial indicators like sales growth, profitability, market share (Nielsen 2007; Bener and Glaister 2010). When the body of research increased, various authors claimed that ISA are often formed not primarily to achieve financial objectives. Instead ISA are, for example, an ideal mode of cooperation to increase organizational learning, which is not measured in financial terms (Kogut 1988; Bener and Glaister 2010). Therefore, literature stresses the *subjective* perception of alliance performance, which is commonly understood as the degree to which each partner company is satisfied with the venture or with their evaluations of the mutually achieved objectives (Geringer and Hebert 1989; Yan and Gray 1994; Glaister and Buckley 1998; Robson et al. 2008; Pak et al. 2009).

Methodological papers and some literature reviews have outlined supplementary possibilities to depict and measure alliance performance. Hence, alliance performance is commonly divided into three principal groups: *(1) financial performance* (e.g., profitability, sales growth), *(2) operational performance* (e.g., stability, contractual changes), and *(3) organizational performance* (e.g., management satisfaction, organizational learning) (Ariño 2003; Robson et al. 2006a, b; Isidor et al. 2012; Christoffersen 2013).

Unfortunately, there has not been a categorization resp. operationalization of alliance performance in behavioral studies following the stated performance dimensions. However, such a breakdown could be useful for further research to understand how alliance performance is interpreted in behavioral research and how it should be applied in upcoming studies. Therefore, we seek to determine which of the three performance dimensions have been applied in behavioral research to capture alliance performance. In particular, the operationalization and measurement should be focused on. Hence, the second research question is formulated as follows:

*RQ2 How is alliance performance operationalized and measured in studies that deal with behavioral constructs?*

Research in the ordinary realm of organizational behavior has acknowledged that, in a regular corporate setting, behavioral constructs relate to business performance and certain variables have been found to be influential (e.g., Chatman et al. 2014; Tognazzo et al. 2017; Pang and Lu 2018). For example, authors analyzed various variables like employee empowerment (e.g., Patterson et al. 2004), turnover, employee training (e.g., Kwon and Rupp 2013), management support resp. aspects of leadership (e.g., Sung and Choi 2014), and organizational culture (e.g., Chatman et al. 2014) to uncover business performance.

Behavioral factors are not easily comparable, since they differ in their nature and their size of influence depends on specific circumstances. Hence, authors have argued that a well-ordered consideration of such constructs could be helpful in clarifying what factors influence business performance and how they do so (e.g., Yuan and Zhou 2015). Research consistently distinguishes among behavioral factors on three levels: the *individual* (e.g., Lam and Schaubroeck 1999; Gupta et al. 2016), *group-related* (e.g., Yuan and Zhou 2015; Gupta et al. 2016), and *organizational level* (e.g., Chatman et al. 2014). When coming to ISA, the need for a differentiated view concerning behavioral constructs is also clear, as studies on the individual (e.g., personal trust, employee training) (e.g., Vaidya and Nasif 2002; Baughn et al. 2011; Ho and Wang 2015), group-related (e.g., management team processes) (e.g., Wai On et al. 2013), and organizational level (e.g., national cultural differences) (e.g., Drouin et al. 2009) are extant. Thus, to get a differentiated overview of which individual, group-related and organizational factors have already been studied in the context of ISA and how alliance performance is affected by these factors, we address the following research questions to reduce the complexity of this fragmented research field:

*What individual (RQ3.1), group-related (RQ3.2), and organizational (RQ3.3) factors have been analyzed to explain the performance of international strategic alliances, and how is alliance performance affected by these factors?*

On the basis of the insights gained from RQ3.1-RQ3.3 we aim to give an overview of the state of the literature in the field of organizational behavior that relates to the performance of ISA and point out specific avenues for future research, as authors stress that this research field should be extended (e.g., Nippa and Reuer 2019).

## Conceptual background

As stated by our study’s aim and the research questions, the primary interest of this systematic literature review is to understand the relation between behavioral factors and alliance performance. Literature suggests to differentiate between the individual, group-related, and organizational level when analyzing behavioral factors of influence (see RQ3.1-RQ3.3) and already acknowledged this differentiation as a commonly used typology (e.g., Meyer et al. 1993). Thus, we incorporate this approach as a *guiding structure for our review*, especially while analyzing the identified articles, but also in the course of depicting avenues for future research.

The deployment of our research questions (in particular RQ3.1-RQ3.3) led to another presumption that influences our study’s approach and its structure. By broadly regarding relevant articles linked to the set research questions, we perceived that a certain multidimensionality of several influencing factors, across the three behavioral levels, appears to be extant. For instance, variables like trust seem to be settled on the individual (e.g., Mohr and Puck 2005; Girmscheid and Brockmann 2010), group-related (e.g., Luo 2002; Krishnan and Martin 2006), and organizational level (e.g., Nisar et al. 2018; Owens et al. 2018). Due to this, we consider that there might exist *categories encompassing factors of influence* that cross at least two and sometimes even all three behavioral levels. Therefore, we also aim to identify such categories by observing the insights that will result from this systematic literature review. Academics of organizational research stress that the identification of homogeneous categories is beneficial and works as a fundamental first step to social theory and research (e.g., Meyer et al. 1993), wherefore we hope to advance the research field linked to ISA and its performance by following this strategy.

**Fig. 1:**
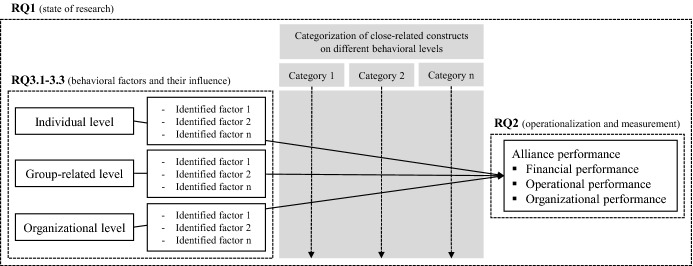
Analytical framework of the review

The pivotal literature-driven approach of this review that targets an extension of the already existing research field of ISA regarding its performance as well as the concept-centric identification of categories across the three behavioral levels are summarized in Fig. 1. This figure also includes the annotation of all research questions, which mirrors our planned procedure comprehensively and functions as a guide throughout the whole review. We aim to fill out this figure by, firstly, identifying all relevant factors of influence that positively or negatively relate to alliance performance and are settled on at least one of the three behavioral levels. Secondly, we further aim to build categories across these three behavioral levels, after profoundly analyzing our derived results. To achieve our set goals, we follow commonly used methodological steps when conducting a systematic literature review that are presented in the following chapter.

## Method and sampling

A systematic review is an organized process of identifying and synthesizing relevant literature to answer specific research questions (Petticrew and Roberts 2006; Snyder 2019). In this study a three-stage analysis was conducted that involves the following steps: (1) relevant scientific databases were identified, (2) the literature was screened using an in-depth structural and content-based analysis, and (3) the identified articles were clustered into concept-centric categories to synthesize the research field (Webster and Watson 2002).

To address the set research questions, we followed a database-driven approach (Hiebl 2021). Such a review strategy considers every available piece of research (Randolph 2009; Snyder 2019) and has been declared as a well-established procedure for performing a systematic literature review (Siddaway et al. 2019). So, the relevant literature is collected using three prominent databases, as done by others before (e.g., Michler et al. 2020; Srivastava et al. 2020): *(1)* *EBSCO (Business Source Premier/EconLit), (2) Web of Science, and (3) Scopus*. These databases have been classified as having been used most often in the sampling procedures of systematic literature reviews and are widely accepted for starting an academic search (Hiebl 2021).

In identifying all relevant literature, we did not limit our search to a particular date of publication in the past (Hiebl 2021). The respective *databases have been analyzed from October 2020 until December 2020*. So all articles published until the end of 2020 are included and those published in 2021 and later have not been included within the framework of this study. The *search was based on a string of 24 terms* directly related to ISA, performance, and organizational behavior (Frank and Hatak 2014; Siddaway et al. 2019; Snyder 2019), as visualized in Table 1.

**Table 1:** Tripartite search string based on keywords

Organizational form	“international strategic alliance” OR “international joint venture” OR “alliance and joint venture” AND
Performance	“perform*” OR “alliance performance” OR “business performance” OR “organizational performance” OR “success” OR “effectiveness” OR “failure” OR “survival” OR “stability” AND
Factor of influence	“organizational behavior” OR “learn*” OR “commitment” OR “trust” OR “job satisfaction” OR “stress” OR “conflict” OR “communication” OR “decision making” OR “lead*” OR “national culture” OR “organizational culture”

As Table 1 shows, “international strategic alliance”, “international joint venture”, and the combination of both terms were included in the first part of the search string. Business practice and the relevant literature stress the significance of international joint ventures (IJV) which are classified as equity-based ISA (DIHK 2012; Isidor et al. 2015). We added this term to the search string because IJV are a commonly used mode of cooperation (Kwok et al. 2019). The second part of the search string addresses the performance of ISA. Despite this general term, several synonyms for alliance performance (e.g., success, effectiveness, survival) were included by deriving them from literature (Dadfar et al. 2014; Kobernyuk et al. 2014). No issue-specific constructs that address factors like individual or marketing performance, which some researchers have discussed as well, were considered for this review. To set up the third part of the search string, we selected textbooks in English and German language that deal with organizational behavior (Hersey et al. 2008; Nerdinger 2012; Martin 2017; Konopaske et al. 2018). The search string was finalized based on the main behavioral constructs described in these textbooks. The search string was created based on subjective assumptions that resulted in 24 terms and should paint a comprehensive picture to answer the research questions.

Each article underwent an initial screening for relevance by two researchers who independently *reviewed the title, abstract, and keywords* and rated it as “in scope” or “out of scope” (Aguinis et al. 2018). Excluding an article always required the consent of both authors (Atkinson et al. 2015). Then, *all articles were read entirely*. As researchers have recommended (Randolph 2009; Atkinson et al. 2015) several *content-based exclusion criteria* were defined to ensure that only studies were included that focus ISA as the type of organizational mode, deal with one of the three types of performance (financial, operational or organizational performance) (Ariño 2003) as a key focus resp. the dependent variable, and include a behavioral construct as an influence factor (conceptual, theoretical or qualitative studies) or independent variable (quantitative studies). Because a lot of ISA-related studies apply quantitative research methods (López-Duarte et al. 2016), the measurement (e.g., scales) of the dependent variable (alliance performance) was also included to answer the second research questions comprehensively. Potential moderator, mediator or control variables that relate to one of the behavioral levels were noted as well. Following Moher et al. (2009) the content-oriented criteria were aggregated to a decision tree (see Appendix 1). *Formal inclusion criteria* were also used throughout the search process (Hiebl 2021). Each article should be in English language, a primary social-scientific study and published in a double-blind peer-reviewed academic journal (Atkinson et al. 2015). Above that, duplicates of identified articles were excluded. This often needs to be done due to the overlapping scope of several databases.

Following this sampling procedure and after merging the three subsamples (EBSCO, Web of Science, and Scopus), a sample size of 145 articles was obtained. Sixteen of these articles were then excluded, since their objectives did not match our research question closely enough. For example, some studies did not deal with alliance performance but with other performance types (e.g., marketing performance) or only implications for ISA were derived, while ISA were not analyzed as an organizational form. In addition, some articles analyzed secondary data which was not obvious while reading only title, abstract and keywords. In the end, the *final sample size of 129 articles* was attained, constituting the foundation for our further analysis.

To structure the 129 identified articles and determine an organizing framework for the review, a *concept-centric approach* was used (Webster and Watson 2002). By using Microsoft Excel each paper was categorized according to “year”, “author(s)”, “article title”, “journal title”, “impact factor”, “method”, “geographic location” and “abstract”, followed by sections addressing the research questions – in terms of the three levels of organizational behavior and their underlying constructs. This approach is used to reduce complexity for presenting data in a manageable structure. Following this, we made use of the just described *concept matrix* (Webster and Watson 2002) to answer our research questions and, thus, it served as a basis for the applied coding cycles. This study follows Saldaña (2016)* to systematically code and analyze the derived qualitative data* which was done within the concept matrix. In order to understand how alliance performance is operationalized and measured (RQ2), we used “structural coding” (Saldaña 2016). This stood to reason because we verified the three existing principal groups (financial, operational, and organizational performance) in our literature sample and thereby followed a deductive approach. For identifying and analyzing behavioral factors that relate to alliance performance (RQ3.1-RQ3.3), we applied two coding cycles. “Descriptive coding” was applied for each included article regarding the factors of influence contained, as this is a pivotal groundwork for any second coding cycle (Saldaña 2016). For the second coding cycle, “pattern coding” was applied. This is an essential step to pull a considerable amount of qualitative data to meaningful units of analysis (Saldaña 2016). The generated units, which represent the factors of influence identified, were allocated to one of the three behavioral levels as shown in Fig. 1 (“identified factor 1-n”). To set up categories across the three behavioral levels another coding cycle was performed. Again, “pattern coding” was used to establish overarching units (Saldaña 2016) that formed our categories and is shown in Fig. 1 as well (“category 1-n”).

## Sample description (RQ1)

**Fig. 2:**
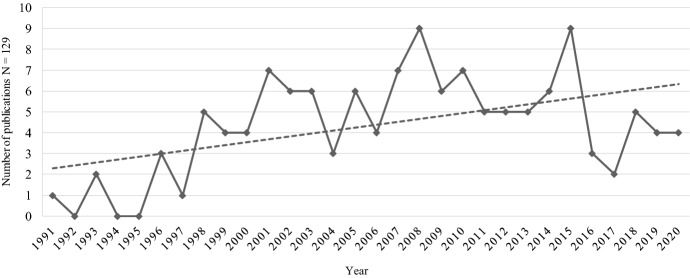
Distribution of publications per year for the period 1991–2020

To answer the first research question, the distribution of publications over time was derived in an initial step, as shown in Fig. 2.

The identified articles were published during a *period of 30 years, starting in 1991*. The body of research is characterized by certain variations in publication per year. However, on average a trend of increasing publications is distinct. The peaks (nine publications in both 2008 and 2015) and thereafter following declines (only three publications in 2004 and only two in 2017) may have been linked to varying levels of interest in these topics during certain time periods. This may be the case, as particularly in the 2000s foundations of ISA were at a constantly high level. Moreover, the formation of IJV increased in 2007, which could have led to intensified research efforts before decreasing in 2009 (Deloitte 2010). Diverging publication processes and special issues could also have led to this distribution of publications. For example, in 2008 and 2015, trust between partnering companies was extensively analyzed (e.g., Fang et al. 2008; Robson et al. 2008; Isidor et al. 2015; Larimo and Le Nguyen 2015). Studies published in 2008 often dealt with national cultural differences (e.g., Kwon 2008; Ozorhon et al. 2008), while studies in 2015 often focused corporate cultural differences (e.g., Gómez-Miranda et al. 2015) between the partnering companies. All of these findings could explain the variations in the publication trends. However, despite the decline in 2016 and 2017, the research domain is still a topic of interest, as publications subsequently increased and remained on a constant level in 2019 and 2020.

**Fig. 3:**
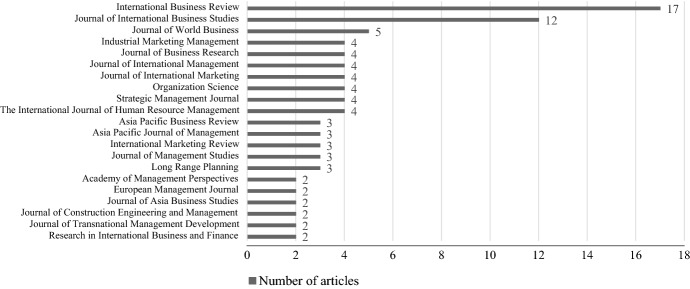
Number of articles published by journals

As RQ1 also focalizes the state of research in terms of academic journals related to this topic, a further glance is attributed to the journals of the derived articles. As already assumed, our chosen database-driven approach is appropriate, since the results indicate that articles from a *total of 61 journals* were included in the sample (see Appendix 2). Had we searched only specific journals, many articles would have been neglected which could have harmed our results. In sum 40 journals contributed a single article to our sample. Figure 3 shows which journals contributed at least two articles to the sample.

As Fig. 3 shows, journals of high importance to this research field are *International Business Review* (contributing 17 articles), *Journal of International Business Studies* (contributing 12 articles), and *Journal of World Business* (contributing 5 articles). Surprisingly, no journal was included that primarily publishes articles directly related to themes of organizational behavior. To validate our sample’s robustness, we conducted a supplementary literature search (Siddaway et al. 2019; Hiebl 2021) using three prominent journals that deal with organizational behavior: Journal of Organizational Behavior, Journal of Vocational Behavior, and European Journal of Work and Organizational Psychology. The additive search led to no new articles that fit our research approach, indicating that our sample of 129 articles presents a comprehensive picture of the relevant literature and should be adequate to answer the research questions. As Tranfield et al. (2003) recommended, we noted down the latest issued impact factors of the journals in which our sample’s articles appeared to assess the sample’s quality. The average impact factor resulted in a numerical value of 3.863, which can be regarded as satisfactory.

Finally, the following distribution of articles regarding the levels of organizational behavior became clear. Most articles address the *organizational level (119 articles)*, followed by articles covering the *individual level (40 articles)*. The *group-related level* is the least represented, since it covers *30 articles*. Of course, one article can focus on more than one of these three levels. For example, in some empirical studies, more than one behavioral construct are analyzed as influencing factors. As for *research methods*, the quantitative approach, used in 75 studies, dominates the research of organizational behavior linked to the performance of ISA. Next, 26 studies are qualitative in nature, followed by 12 studies that can be categorized as conceptual. Furthermore, 16 studies used a mixed-method approach.

## Content-related results

### Operationalization and measurement of performance (RQ2)

RQ2 considers the operationalization and measurement of alliance performance in studies that deal with behavioral construct. As a groundwork another glance has to be attributed to the already existing literature that reveals three main categories of alliance performance (Ariño 2003; Robson et al. 2006a, b; Christoffersen 2013): *(1) financial performance, (2) operational performance, and (3) organizational performance.* First, we determine whether these three categories have been addressed at all by academics who have dealt with behavioral research linked to ISA. Then the operationalization of performance constructs within one category and their measurements will be dealt with.

The literature’s use of the first category, *financial performance,* has been marginal, because only 5 of the 129 articles chose this approach (Jennings et al. 2000; Luo 2002, 2009; Fang et al. 2008; Li et al. 2012). Our analysis shows that measures like return on assets (ROA) (Li et al. 2012), return on investment (ROI) (Luo 2002, 2009), and sales growth (Luo 2002; Fang et al. 2008; Li et al. 2012) have functioned as operationalizations of alliance performance. Some articles, such as Li et al. (2012), used joint measures (a combination of ROA and sales growth) to capture alliance performance. Academics usually drew on internal company data or used databases to build their measures (Luo 2002; Fang et al. 2008).

The *operational performance* category was addressed more often than the financial performance category. Generally speaking, two different operationalizations were primarily applied: *(in)stability* and *survival*. (In)stability, which relates to relational changes in the control structure and decision rules in an ISA (Inkpen and Beamish 1997) and is also often described as “longevity” (Salk and Shenkar 2001), was transferred to an item scale by Fang and Zou (2010) that other researchers have used and adapted (e.g., Isidor et al. 2015). The scale asks respondents for the extent to which the ownership/management structure of their ISA had changed during the previous three years in ways that were not originally planned (Fang and Zou 2010). Some authors also applied (in)stability as a dichotomous variable (e.g., Steensma et al. 2008), coding the dependent variable 1 if the cooperation converted to a wholly owned subsidiary and 0 if it remained an ISA. (In)stability was also captured as a target construct by several qualitative or conceptual studies (Salk and Shenkar 2001; Schuler 2001; Ott 2015; Owens et al. 2018). Coming to survival resp. termination/failure of an ISA (Pajunen and Fang 2013; Dadfar et al. 2014), qualitative studies in particular used this term as their target construct (Ariño and La Torre 1998; Hambrick et al. 2001; Watts and Hamilton III 2007; Pajunen and Fang 2013).

The last category, *organizational performance*, is the most frequently applied. Various operationalizations have been used that will be presented in the following. First of all, the closely-related constructs *degree of goal fulfilment* and *management satisfaction* were applied in several studies. Some authors used or adopted the same scale of measurement (e.g., Lyles and Salk 1996) and described the outcome variable as either “degree of goal fulfilment” (e.g., Baughn et al. 2011) or “management satisfaction” (e.g., Robson et al. 2006a, b; Park et al. 2012), while others combined these two ways of measuring alliance performance (e.g., Ozorhon et al. 2008; Wai On et al. 2013; Larimo and Le Nguyen 2015; Kwok et al. 2019; Huang and Chiu 2020). However, degree of goal fulfilment can be particularly classified as the achievement of the partnering companies’ goals. These can be objective/financial goals (e.g., sales) or subjective/non-financial goals (e.g., quality of relationship) (Thuy and Quang 2005; Thuc Anh et al. 2006; Chen et al. 2009; Ho and Wang 2015; Liu et al. 2017, 2020). Management satisfaction outlines how the partnering companies and the general manager(s) of an ISA evaluate the overall alliance’s performance (Dhanaraj et al. 2004). Generally speaking, management satisfaction is measured by aspects like profitability and human resource productivity (Lin and Germain 1998; Demirbag and Mirza 2000; Gong et al. 2001; Lane et al. 2001; Zeybek et al. 2003; Lin 2007; Mohr and Puck 2007; Lin and Wang 2008; Zhan and Luo 2008; Pak et al. 2009; Yan et al. 2010; Damanpour et al. 2012; Nisar et al. 2018). Finally, dozens of measurement scales were used to capture this type of alliance performance (e.g., long-term satisfaction, satisfaction relative to competitors, etc.) (Lane et al. 2001; Mohr and Puck 2005; Farrell et al. 2011). However, the two most prominent measures are the scales of Lyles and Salk (1996) and Dhanaraj et al. (2004), which were used or adopted by a lot of researchers (e.g., Robson et al. 2006a, b; Farrell et al. 2011; Damanpour et al. 2012).

*Organizational learning* and *knowledge transfer/acquisition* have to be considered as well. Organizational learning describes a process of translating individual capabilities into organizational capabilities by interpreting, integrating, and institutionalizing knowledge (Crossan and Berdrow 2003; Liu and Zhang 2014), which is one of the central aims in creating an ISA (Parmigiani and Rivera-Santos 2011). The literature has emphasized that organizational learning is decisive to be competitive, so it is considered as a proxy for alliance performance (Liu and Zhang 2014). Therefore, authors used terms like “learning effectiveness” (Vaidya and Nasif 2002) and “inter-partner learning” (Hamel 1991) as their target construct. The transfer or acquisition of knowledge can also be regarded as expressing alliance performance (Park et al. 2009; Park 2010). Therefore, the ability of the partner companies to absorb knowledge from each other (Martin and Emptage 2019) or the speed with which knowledge is transferred (Khan et al. 2015) have been regularly measured as alliance performance. Several researchers adopted Dhanaraj et al.'s (2004) scale, which has been used mainly to measure management satisfaction, to capture the transfer/acquisition of knowledge (Park and Vertinsky 2016; Minbaeva et al. 2018), since some of its items relate to the internalization of knowledge. However, other scales have also been used and/or adopted (e.g., Khan et al. 2015).

We emphasize that all performance dimensions resp. operationalizations have been considered to analyze the articles in our sample and to answer the research questions (particularly RQ3.1-RQ3.3). Hence, we use only the term “alliance performance” in the following, when referring to the various ways the articles have expressed alliance performance.

### Individual factors (RQ3.1)

To begin with behavioral factors that have been analyzed to explain the performance of ISA, the literature that is linked to the individual level, highlights *personal trust* as one of the most striking factors. Personal trust refers to a connection among actors that constitutes the pattern of interactions in an ISA (Robson et al. 2019) and determines whether individuals can rely on each other (e.g., Mohr and Puck 2005; Girmscheid and Brockmann 2010). Research states that this form of trust is central to alliance performance (Currall and Inkpen 2002; Styles and Hersch 2005; Brouthers and Bamossy 2006; Girmscheid and Brockmann 2010; Robson et al. 2019). Some studies have used this factor as a moderating variable and confirmed its positive impact (e.g., Mohr and Puck 2005). However, Robson et al. (2019) found that the influence of personal trust in an ISA should be depicted as having an inverted U-shape, as the positive effect on alliance performance decreases after reaching a certain threshold. The authors explained that, because of inertia in the long run, personal trust might not have the positive effect that it has in the beginning.

The literature has stressed the *organizational commitment* of individuals who are part of an ISA (e.g., Turpin 1993; Jennings et al. 2000) and has found it influential in maximizing alliance performance (Jennings et al. 2000; Vaidya and Nasif 2002). Related to organizational commitment and personal trust is *relational capital*, which is an accumulated factor consisting of several variables like commitment, trust, friendship, and mutual understanding that reside at the individual level (Thuy and Quang 2005). An article that dealt with this factor worked out that relational capital functions as a positively mediating variable (Thuy and Quang 2005). It was also used as a moderating variable that supported positive relationships concerning alliance performance (e.g., Ho and Wang 2015). In this regard, closely-related factors, such as *personal interaction* and *communication*, should be highlighted as well. Some authors have even depicted such factors as part of relational capital (e.g., Lo et al. 2016) and argued that frequent interactions between individuals in an ISA and fluent communication lead to positive performance outcomes. The literature has also emphasized that, when individuals are not willing to share information or do not communicate appropriately, alliance performance is inhibited (Cyr and Schneider 1996), so managers should strive to solve such tensions quickly.

Another important factor is *individual learning* (Inkpen and Dinur 1998; Schuler 2001; Iles and Yolles 2002; Thuc Anh et al. 2006). Research revealed a positive influence of this variable and states that learning occurs on all three levels (individual, group-related, organizational level). However, learning starts with the individual in meetings, visits, tours, personnel transfer to the ISA (e.g., expatriation), etc. (Inkpen and Dinur 1998; Schuler 2001). Beyond that, literature has found that an executive’s *prior knowledge/experience* regarding the management of an ISA, which can be described as an expanded learning process, is influential for alliance performance (e.g., Ng et al. 2007), as upcoming learning processes will be speeded up (e.g., Park and Harris 2014). Further, the *learning capability* of an individual*,* which can be described as the ability to codify and articulate knowledge (Minbaeva et al. 2018), was observed. Nevertheless, Likhi and Sushil (2013) as well as Minbaeva et al. (2018) were not able to support their assumption that this factor positively influences alliance performance. Liu and Zhang (2014) highlighted in their qualitative study that such internalization and externalization of knowledge by the individual leads to learning outcomes, but they also echoed the results of Likhi and Sushil (2013) and Minbaeva et al. (2018), by accentuating that it always requires effective mechanisms, implemented by the ISA, that help to translate individual learning into organizational learning. So, individual learning capabilities do not guarantee effective learning and should be embedded into steady processes established by the ISA. Finally, the literature has ascertained a positive influence of the variable *intent to learn*, which is the step before the actual learning (Park et al. 2009; Park and Vertinsky 2016).

*Employee training* has also been considered as a factor of huge meaning (Cyr and Schneider 1996; Lyles and Salk 1996; Child and Yan 2003; Park et al. 2009; Park 2010; Baughn et al. 2011). This term refers to training in technical and managerial skills, but skills like conflict resolution and foreign language skills are also trained since ISA are always embedded in a cross-cultural setting (Cyr and Schneider 1996). Research has unanimously argued that individual training affects knowledge acquisition resp. alliance performance (Lyles and Salk 1996; Park et al. 2009; Park 2010). However, Baughn et al. (2011) opined that cross-cultural training is most influential because ISA require that people from at least two nations cooperate. Some authors have also contended that steady investment in training programs should take place which will enable employees to acquire knowledge (Lyles and Salk 1996; Child and Yan 2003; Thuc Anh et al. 2006). Furthermore, Lyles and Salk (2007) brought into focus training that aim to harmonize the capabilities of employees, whose skills may differ markedly from the employees of the partner company. Implementing adaptive trainings could help to ensure the effective flow of knowledge between these employee groups (Lyles and Salk 2007).

Other articles have examined the (general) manager of an ISA in detail. For example, Mohr and Puck (2007) observed *occupational stress* in this regard. Their empirical analysis found that a high level of occupational stress relates to low alliance performance. Their argumentation was based on *role conflicts* for managers of ISA, since at least three parties (the ISA and the partnering companies) address these individuals. Such conflicts can lead to different expectations and roles to be occupied by the managers, which may be incompatible with the person’s own needs (Mohr and Puck 2007). Gong et al. (2001) analyzed role conflicts in this context, although they disagreed with Mohr and Puck's (2007) results, as they showed that role conflicts positively influence alliance performance. They argued that the conflicting objectives that multiple parties convey to the managers may motivate these individuals to increase their level of effort, which would have positive influence on alliance performance. Contrary to Gong et al. (2001), Li et al. (2002) supported Mohr and Puck's (2007) insights and also claimed that role conflicts can result in occupational stress and negatively influence alliance performance (Li et al. 2002). Clearly, research on this topic is inconsistent and not finalized.

### Group-related factors (RQ3.2)

The factor most often considered on the group-related level is *inter-personal conflicts* (Fey and Beamish 1999; Tsang et al. 2004; Pak et al. 2009). In general, research has agreed that inter-personal conflicts between individuals in an ISA (e.g., from differing opinions) worsen alliance performance (e.g., Li et al. 2002; Pak et al. 2009; Pajunen and Fang 2013). Tsang et al. (2004) concluded that the intensity, not the frequency, of conflicts has a negative impact on alliance performance. Dealing with conflict management by regarding interactions of at least two individuals within an ISA, research has started to analyze this factor by including it in a mediation relationship. For instance, Thuy and Quang (2005) outlined that conflict management can be seen as an influencing factor when relational capital (see RQ3.1) is included as a mediating variable. Liu et al. (2020) incorporated conflict management as a moderator in their study and clarified this factor further by distinguishing among three approaches to handle conflicts: cooperative, competitive, and avoiding. The authors determined that the negative effect of national cultural differences on alliance performance is mitigated only by adopting the cooperative approach to conflict management (Liu et al. 2020).

The next focus is on *(management) teams* in an ISA. Analyzing the sample of articles made evident that factors like trust (e.g., Currall and Inkpen 2002; Luo 2002; Girmscheid and Brockmann 2010; Wai On et al. 2013), commitment (e.g., Dadfar et al. 2014; Liu and Zhang 2014; Owens et al. 2018), and communication (e.g., Zeybek et al. 2003; Choi et al. 2010) are extant on the group-related level as well. Furthermore, these factors help to guarantee the functioning of (management) teams in ISA, as their activities regularly relate to alliance performance.

Starting with *trust* among *members of (management) teams* as an influencing variable, research has consistently found that this factor is positively related to alliance performance (e.g., Currall and Inkpen 2002; Girmscheid and Brockmann 2010). Luo (2002) emphasized that collectivist cultures tend to rely on personal links resp. trust in their (management) team(s), so ISA that are initiated by companies in individualist nations should be especially aware of this finding when they cooperate with companies from collectivist nations. Wai On et al. (2013) included this type of trust as a mediating variable in their study and found that the relationship between the independent variables (e.g., national culture) and alliance performance was mediated by this factor. Thus, trust in teams is particularly important when companies whose national backgrounds highly differ are cooperating in an alliance.

*Commitment* as it relates to (management) teams requires a certain distinction. On the one hand, academics like Owens et al. (2018) as well as Liu and Zhang (2014) have depicted commitment as a relational variable that subsists between individuals of a (management) team, guiding their interactions and influencing alliance performance. This type of commitment has often been designated as affective commitment between the members of a team, as it describes a psychological attachment (Owens et al. 2018). On the other hand, Dadfar et al. (2014) argued that teams in an ISA have always been seen as an aggregated body of operating managers who are mutually more or less committed to the ISA. This accumulated commitment concerning the venture itself can influence alliance performance as well.

Considering (management) teams of ISA even further, researchers like Owens et al. (2018) have outlined *social interaction processes* like individuals’ ability to influence their colleagues. Therefore, by influencing partners’ opinions and building consensus through relational persuasion, conflicts may dissolve faster and joint decisions may be attained more quickly. In addition, Inkpen and Dinur (1998) stated that social interaction processes are essential to achieve satisfactory learning outcomes and enhance alliance performance. *Communication* inside the (management) teams has to be named alike. Research has concentrated on the frequency (Zeybek et al. 2003; Choi et al. 2010) as well as on the efficacy of team communication (Choi et al. 2010). The higher these dimensions, the better an ISA will perform.

Another topic of discussion has been the *composition of (management) teams*. Research has recognized demographic (e. g., functional background, language abilities) and psychological attributes (e.g., values, norms). If the (management) team is a combination of managers from at least two companies, compositional gaps regarding these attributes can emerge, leading to substantive conflicts and harming alliance performance (Hambrick et al. 2001; Drouin et al. 2009). Li et al. (1999) showed that a mismatch in the management team structure can lead to role conflicts, which negatively impact alliance performance (see RQ3.1). However, Hambrick et al. (2001) concluded that a moderate level of conflict that results from the team’s composition can have a positive effect on alliance performance because fertile discussions between the team members are likely to occur. But, the authors described this relationship as taking an inverted U-shape such that the greater these compositional conflicts, after a certain threshold, the lower alliance performance will be.

Factors that are linked to *leadership* have barely been addressed and only conceptual and qualitative studies have been conducted, which reflects the incomplete nature of this research facet. Researchers have agreed only that effective leadership is vital and impacts alliance performance (Li et al. 1999; Likhi and Sushil 2013). Demir and Söderman (2007) argued that powerful leadership improves individual learning, which is central to alliance performance (see RQ3.1). However, it is fundamentally important to deepen knowledge in this regard. Ordinary research of organizational behavior stresses the significance of several leadership aspects (e.g., leadership style, personality traits, decision making) when observing organizational performance (e.g., Howell et al. 2005; Kiss et al. 2021). Thus, it seems fruitful to apply the already gained knowledge to the exceptional organizational context of ISA.

### Organizational factors (RQ3.3)

Central themes of interest on the organizational level refer to *inter-organizational learning* (Hamel 1991; Ariño and La Torre 1998; Demirbag and Mirza 2000; Lane et al. 2001; Schuler 2001; Beamish and Berdrow 2003; Child and Yan 2003; Farrell et al. 2011; Park and Vertinsky 2016) and *knowledge management* (Inkpen 1998; Inkpen and Dinur 1998; Likhi and Sushil 2013; Liu and Zhang 2014). For example, research has analyzed factors like *intent to learn* (Park 2010; Park and Vertinsky 2016; Martin and Emptage 2019) and *learning orientation* (Mehta et al. 2006; Farrell et al. 2008, 2011) of the ISA. Both factors have been identified as having a positive effect on alliance performance (Park 2010; Farrell et al. 2011; Park and Vertinsky 2016). When coming to knowledge management various aspects like the type of knowledge (Acharya et al. 2020), knowledge protection (Ho and Wang 2015), openness to share knowledge (Liu and Zhang 2014), and knowledge-based resources (Kim et al. 2011) have been studied, although the topics addressed most often are *knowledge transfer/acquisition*. In general, the literature has agreed that a fluent transfer of knowledge between the partnering companies in an ISA, but particularly the ability to acquire this transferred knowledge amplifies alliance performance since this is a central reasons for most ISA having been established (Turpin 1993; Griffith et al. 2001; Pak et al. 2009; Lee et al. 2011; Liu and Zhang 2014).

However, some studies have had other insights. Mohedano-Suanes and del Mar Benavides-Espinosa (2013) stated that knowledge acquisition might lead to changes in the extent of control one of the partner companies exercises over the ISA, which diminishes the chance of survival. Moreover, authors like Dhanaraj et al. (2004) have found no direct relationship between knowledge transfer and alliance performance. Such voices have led to the incorporation of another factor, *absorptive capacity (ACAP)*, which has been included as an independent (Lyles and Salk 2007), but also a mediating (Lane et al. 2001) and, most notably, a moderating variable (Fang and Zou 2010; Kim et al. 2011; Zhang et al. 2018) in several studies. ACAP can be understood as the collective ability to recognize the value of new information, assimilate it and apply it in a company (Cohen and Levinthal 1990). Research has found that ACAP is a key construct in explaining alliance performance (Lane et al. 2001; Lyles and Salk 2007; Kim et al. 2011), so ACAP must be included in further research.

One of the most frequently considered topics on the organizational level is linked to culture, as national and organizational cultural differences have been observed to explicate alliance performance. Most researchers have built on Hofstede's (1983) dimensions to analyze the differences in the *national cultures* of partnering companies that aim to create an alliance project (Barkema and Vermeulen 1997; Ott 2015; Pauluzzo and Cagnina 2019). The literature has indicated that significant differences in companies’ national cultural dimensions lead to a decline in alliance performance (Barkema and Vermeulen 1997; Pothukuchi et al. 2002; Pak et al. 2009; Ott 2015; Pauluzzo and Cagnina 2019; Liu et al. 2020). However, a few studies have found no relationship between differences in partnering companies’ national culture and alliance performance (Avny and Anderson 2008; Bener and Glaister 2010). Beyond that, some academics have claimed that national cultural differences are not that important for an ISA in corporate practice as it was stated in the literature until now (e.g., Li et al. 2016). Though, differences in the *organizational culture* have been classified as highly influential (Ozorhon et al. 2008). Several studies have analyzed organizational cultural differences and determined that alliance performance can be harmed by such differences, primarily because of emerging conflicts and relational problems (Pothukuchi et al. 2002; Bener and Glaister 2010). Moreover, corporate cultural compatibility has a positive impact on alliance performance, which has been confirmed by academics alike (Vaidya and Nasif 2002; Park et al. 2009; Park 2010). Differences that reside in the organizational culture have been based on various conceptualizations. For example, Low et al. (2020) conceived organizational culture by deploying elements like “market orientation” and “teamwork orientation”. Contrary to this, Gómez-Miranda et al. (2015) preliminary focused on elements like “focus on workforce” and “focus on management interest” to capture organizational culture. This differences show that the results that are linked to organizational cultural differences are hardly comparable.

Often resulting from cultural differences, *inter-organizational conflicts* should be monitored likewise (Lyles and Salk 1996, 2007; Li et al. 2016; Pauluzzo and Cagnina 2019). The conflicts that occur between the partnering companies, which are usually control-related issues (Kauser and Shaw 2004), have been addressed extensively, albeit without reaching a conclusion whether such conflicts are beneficial or detrimental to alliance performance. Many articles have confirmed a negative influence of inter-organizational conflicts on alliance performance (Eroglu and Yavas 1996; Ramaseshan and Loo 1998; Demirbag and Mirza 2000; Steensma and Lyles 2000; Kauser and Shaw 2001, 2004; Steensma et al. 2008; Pak et al. 2009; Hsieh et al. 2010), while several other studies have found no significant relationship at all (Lyles and Salk 2007; Farrell et al. 2011). Some authors, such as Demirbag et al. (2003), have even claimed that inter-organizational conflicts should be welcomed, as they can result in improved processes. Clearly a certain disunity concerning this factor remains.

One of the major topics on the organizational level is i*nter-partner trust.* However, compared to personal trust, inter-partner trust describes the partner companies’ intention to accept vulnerability based on positive expectations of the other company (Luo 2002; Krishnan and Martin 2006). Since the beginning of research in this domain, academics have dealt with the topic of inter-partner trust (e.g., Ramaseshan and Loo 1998) and still analyzed it in the recent past (e.g., Martin and Emptage 2019). The literature has unitary argued that trust between the partnering companies is essential – especially for the long-term success (Ariño and La Torre 1998; Cullen et al. 2000). Numerous empirical studies have tested the relationship between inter-partner trust and alliance performance and have consistently found a positive relationship (Kauser and Shaw 2001; Luo 2002; Demirbag et al. 2003; Kauser and Shaw 2004; Nielsen 2007; Kwon 2008; Lin and Wang 2008; Robson et al. 2008; Park et al. 2009; Bener and Glaister 2010; Park 2010; Mohedano-Suanes and del Mar Benavides-Espinosa 2013; Dadfar et al. 2014; Larimo and Le Nguyen 2015; Ali and Khalid 2017; Nisar et al. 2018; Owens et al. 2018).

*Inter-organizational commitment* is understood as a partner’s intention to continue the alliance relationship (Cullen et al. 2000). Although a few articles did not support the positive assumed relationship between inter-organizational commitment and alliance performance (e.g., Luo 2009; Farrell et al. 2011), many authors have validated this relationship empirically (Ramaseshan and Loo 1998; Lin and Germain 1999; Cullen et al. 2000; Demirbag and Mirza 2000; Kauser and Shaw 2001, 2004; Larimo and Le Nguyen 2015).

The literature on *communication* between the partner companies has usually focused on the quality of communication (Kauser and Shaw 2004; Robson et al. 2006a, b) or the level of information-sharing (Kauser and Shaw 2001, 2004). These studies confirmed that a high level of inter-firm communication improved alliance performance. In addition, some studies tested inter-partner communication as a moderating (e.g., Mohr and Puck 2005) or independent variable (e.g., Ramaseshan and Loo 1998) and acknowledged a positive impact. Besides the issue of communication, Nielsen (2007) confirmed that protective behavior by the partner companies (e.g., no information-sharing) negatively relates to alliance performance. Martin and Emptage (2019) supported this assumption by making clear that the more interactions in an ISA take place, the more alliance performance will increase.

Other studies have focused on *mutual dependency* between the partner companies of an ISA. Taking several academic voices into consideration, it becomes obvious that mutual dependency is strengthening the inter-organizational relationship and therefore improving alliance performance (Kauser and Shaw 2001, 2004; Farrell et al. 2011). Hsieh et al. (2010) additionally confirmed that mutual dependency between partners of an ISA does not lead to a higher perception of risk, which supports the statement of Robson et al. (2006a, b) that mutual dependency is negatively related to insecurity in an ISA. Furthermore, the literature has argued that active *(managerial) support/involvement* by the partner companies positively impacts alliance performance (Steensma and Lyles 2000; Lane et al. 2001; Lyles and Salk 2007; Park et al. 2009; Park 2010) and that a similar *management style* of the partner companies has a positive influence as well (Larimo and Le Nguyen 2015).

Themes of staffing like *expatriation management* are vital to name as well. All studies dealing with expatriation management observed knowledge acquisition as alliance performance (Lyles and Salk 1996; Thuc Anh et al. 2006; Park et al. 2009; Park 2010). The use of expatriates (e.g., delegated employees in an ISA) has been found to be positively associated with the level of knowledge acquired from the (foreign) partner company, because the gathered knowledge can be directly forwarded to the company, which had to enter a foreign country (Thuc Anh et al. 2006; Park et al. 2009). Park (2010) added the insight that long-term personnel transfers are more helpful than short stays, as a long stay opens the possibility for frequent interactions, supporting knowledge acquisition (Lyles and Salk 1996). Focusing on *staffing* in general, academics have stated that emphasis should be placed on hiring young employees who easily acclimatize in the special structure of an ISA (e.g., Cyr and Schneider 1996). In addition, some articles have addressed the issue of selecting general managers. A wide variety of antecedents can influence the behavior of these managers, who are appointed by either the partner company that remains in its home country or the partner company that enters a foreign country of operation (e.g., Lin 2007). Therefore, such a decision must be well thought out.

Finally, individuals’ *organizational identification* with the ISA was also analyzed. Research made clear that members of an ISA tend to identify more with their original company than with the ISA (e.g., Li et al. 2002). However, in some cases, one of the partner company’s identity became dominant and the other’s identity vanished (e.g., Salk and Shenkar 2001). It seems, then, that a mutually created organizational identity of an ISA is unlikely, which could harm alliance performance.

## Summary and discussion

This comprehensive and systematic literature review was conducted to shed light on the effect of behavioral factors on the performance of ISA. Among its findings, it became clear that this *research domain is steadily attracting researchers*, since the average number of publications (129 articles published from 1991 to 2020) is increasing (see Fig. 2). However, the volume of publications has varied, and a wide range of authors/journals have addressed this domain, which has led to a fragmented body of research. A number of 61 journals have published articles in this area and surprisingly, no journal, primarily dealing with themes in the realm of organizational behavior, has been identified. Journals that address strategic/international management have dominated, so that hitherto a one-sided perspective has been set to consider these behavioral factors of influence. This outcome is accompanied by the fact that most articles in our sample correspond to the organizational level (119 articles) since aspects of this level (e.g., organizational culture) are closely-related to the themes that are regularly discussed in journals of strategic/international management (e.g., Strese et al. 2016). However, by applying a behavioral lens, this research domain could benefit from – and be extended due to – theoretical and methodological approaches that root in the literature of organizational behavior and, therefore, may differ compared to those of strategic/international management. In most cases, the articles of our sample used quantitative approaches, as only 26 of the 129 articles in the sample are qualitative in nature. So, explanatory studies are still scarce, but should be conducted more frequently, since some topics have ostensibly not yet been analyzed thoroughly (e.g., on the group-related level). Qualitative research has the strength to explore complex issues that are apparent in (business) practice (e.g., the analysis of individual perceptions) which would empower academics to understand certain factors in great detail (Coyle 2016; Mey and Mruck 2020). Furthermore, the derived sample size of 129 articles demands additional discussion, since this count is surprisingly high. Due to statements in literature (e.g. Nippa and Reuer 2019; Srivastava et al. 2020) we assumed a much lower figure, as research is stressing the need for including behavioral factors in studies that deal with ISA, wherefore academics called for doing it. Contrary to this, a certain amount of research already appears to exist. However, to weaken this statement somewhat, we have to consider that our literature sample contains many articles that have included behavioral factors as moderating or mediating variables. These variables influence alliance performance only indirectly. Hence, several authors mainly dealt with factors of other research areas (e.g., functional diversity, distributive justice) in their studies (e.g., Mohr and Puck 2005; Luo 2009). Nevertheless, due to our defined inclusion criteria, these articles have been included as well and as a result, the literature sample became much higher than expected.

The results concerning our second research question, which addresses the operationalization and measurement of alliance performance, showed that the assumed categorization, stated in literature (Ariño 2003; Robson et al. 2006a, b; Christoffersen 2013), is apt. The *first category (financial performance)* is represented the least in our literature sample, as only five articles address this category, perhaps because ISA are often created to learn from or with the partnering company (Schuler et al. 2004; Parmigiani and Rivera-Santos 2011), wherefore a detailed listing of financial data is not necessary. Moreover, ISA are also often formed for only a limited period of time (Pett and Dibrell 2001), so long-term financial data is not tracked. The *second category (operational performance)* was addressed in our sample somewhat more often and was expressed in terms of either (in)stability (e.g., Steensma et al. 2008; Fang and Zou 2010) or survival (e.g., Pajunen and Fang 2013; Dadfar et al. 2014). Most of the derived articles referred to the *third category (organizational performance)*, which is segmented into the sub-categories “degree of goal fulfilment, management satisfaction” and “organizational learning, knowledge transfer/acquisition” – all measured subjectively using questionnaire surveys in quantitative studies (e.g., Yan et al. 2010; Damanpour et al. 2012; Park and Vertinsky 2016; Minbaeva et al. 2018; Nisar et al. 2018). The presence of so many subjective performance indicators was unexpected, as research on ordinary organizational behavior, that has dealt with business performance, has commonly used financial data like operating cash flow and ROA (e.g., Kwon and Rupp 2013; Sung and Choi 2014; Chatman et al. 2014). However, this is accompanied by the above stated facts that ISA do not often show up all usually tracked financial data. Finally, some studies saw organizational learning as a substitute for alliance performance (Liu and Zhang 2014), but it was also used as an independent variable in other studies. Our review outlined that this variable, and knowledge transfer/acquisition, are often regarded as independent variables that influence different types of alliance performance (e.g., Farrell et al. 2011; Lee et al. 2011). This finding reveals a certain ambiguity of constructs that are related to learning in an alliance setting.

**Fig. 4:**
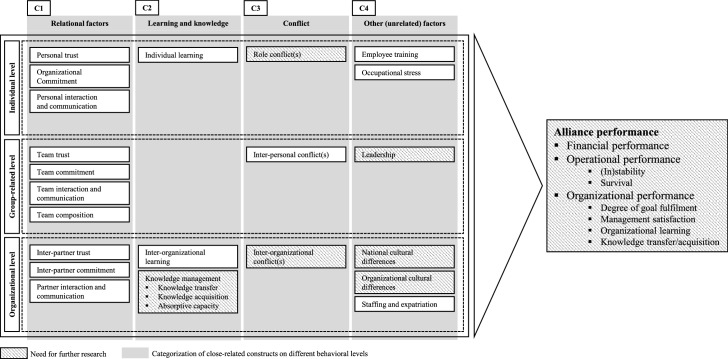
Categorization of influencing factors across the three behavioral levels

The results concerning the factors of influence (see RQ3.1-RQ3.3) show that research in this area can be categorized as assumed in chapter 3. Following Webster and Watson’s (2002) recommendations for presenting the results of a review, Fig. 4 was created by extending Fig. 1 and, thus, it was filled out with the insight generated by our review. It presents the identified categories across the three behavioral levels and the embedded factors therein that influence alliance performance: relational factors (C1), learning and knowledge (C2), conflict (C3), and other (unrelated) factors (C4). We identified these four categories, since by analyzing the presented results, it cleared out that in fact a certain *multidimensionality of several behavioral factors*, across at least two (and sometimes even all three) behavioral levels, is extant.

The category “*relational factor*s” includes variables like trust, which is settled on the individual, group-related, and organizational level, although this factor is interpreted differently on each level. On the individual level, some researchers have understand trust as a connection among actors that indicates whether they can rely on each other (e.g., Mohr and Puck 2005; Girmscheid and Brockmann 2010). However, on the organizational level, trust is seen as the intention to accept vulnerability based on positive expectations one assigns to the company with which one partners (Luo 2002; Krishnan and Martin 2006), which is a different understanding than that on the individual level. The factors “commitment” and “communication” are also present on all three behavioral levels and are understood heterogeneously. Nevertheless, the high occurrence of all of the named factors can be declared as positively influential on alliance performance (see RQ3.1-RQ3.3).

The category *“learning and knowledge”* considers particularly the individual and organizational level. The literature has stated that learning starts with individuals but also occurs on the group-related and organizational level (Inkpen and Dinur 1998; Schuler 2001), although this review found no articles on the group-related level. Learning and related factors have been shown to be influential on alliance performance (e.g., Farrell et al. 2011; Park and Vertinsky 2016; see RQ3.1 and RQ3.3). Articles that addressed the organizational level took a step farther by analyzing knowledge (management), notably in terms of knowledge transfer, knowledge acquisition, and ACAP. In particular, knowledge acquisition is linked to obscurities since it does not always relate to positive performance outcomes (e.g., Dhanaraj et al. 2004), wherefore ACAP is declared as a construct of further academic interest (see RQ3.3).

The category *“conflict”* also covers all three behavioral levels. Starting with the individual level, role conflicts within individuals, mostly managers, in an ISA have been observed (Gong et al. 2001; Li et al. 2002; Mohr and Puck 2007). This conflict manifestation has to be isolated from inter-personal conflicts (group-related level) and inter-organizational conflicts (organizational level). Research has regarded inter-personal conflicts as tensions between individuals, typically resulting from differing opinions in a (management) team (e.g., Li et al. 2002; Pak et al. 2009; Pajunen and Fang 2013), whereas inter-organizational conflicts occur between the partnering companies, usually in terms of control-related issues (Kauser and Shaw 2004). Academics have consistently emphasized that inter-personal conflicts worsen alliance performance (see RQ3.2), but it is not yet clear, whether role conflicts and inter-organizational conflicts positively or negatively impact the performance of ISA (see RQ3.1 and RQ3.3).

The final category, labelled as *“other (unrelated) factors”*, covers all remaining factors that are not interlinked between each other. On the one hand, one has to name the following factors that positively influence alliance performance: employee training (individual level) (e.g., Park 2010), leadership (group-related level) (e.g., Likhi and Sushil 2013), staffing, and expatriation (management) (organizational level) (e.g., Thuc Anh et al. 2006). On the other hand, these negatively influencing factors have to be mentioned as well: occupational stress (individual level) (e.g., Mohr and Puk 2007), national (Liu et al. 2020), and organizational (e.g., Bener and Glaister 2010) cultural differences (organizational level) (see RQ3.1-RQ3.3).

Considering everything discussed, we conclude that three dominant categories (relational factors, learning and knowledge, and conflict) can be recognized that cross at least two behavioral levels. Furthermore, additional factors, which are not interlinked, have been subordinated into a non-specifically labelled category (other (unrelated) factors). To provide a concluding overview, we created Table 2 which recognizes the just described categories. Every identified article is subordinated to at least one or even more categories, since one article might include different factors that are located in different categories. Furthermore, this table also includes additional information regarding the analyzed articles (“author(s) and year of publication”, “title”, “method”, “main outcome(s)”), in order to grasp the main insights at one glance. Moreover, it is vital to state that some factors (e.g., leadership) are not distinctly separable regarding the three behavioral levels (individual, group-related, organizational level) as well (e.g., behavior of a leader vs. interaction with subordinates), which is illustrated by the dotted lines in Fig. 4.

**Table 2:** List of all articles analyzed, classified by the four established categories

Author(s) and year of publication	Title	Method	Category	Main outcome(s)
Acharya et al. (2020)	Race to learn: knowledge char-acteristics and resource structure	Mixed	C2	The results show that parties in IJV have low tendencies to acquire tacit and specific knowledge, but when the resource is complementary, it stimulates the learning race
Huang and Chiu (2020)	A knowledge tension perspective on management control and performance in international joint ventures	Quantitative	C2	This study highlights the moderating role of knowledge tension on the relationship between management control and the partner companies’ satisfaction with IJV performance
Liu et al. (2020)	Impact of culture differences on performance of international construction joint ventures: the moderating role of conflict management	Quantitative	C3; C4	Alliance performance declines with a high degree of national cultural differences. This negative effect is mitigated by adopting the cooperative conflict management approach
Low et al. (2020)	Organisational culture of malaysian international construction organisations	Quantitative	C4	Client orientation is the most striking element regarding the organizational culture, when considering alliance performance, and a competitive culture is critical
Kwok et al. (2019)	Interactive effects of information compaexchange, relationship capital and environmental uncertaintyon international joint venture (IJV) performance: an emerging markets perspective	Quantitative	C1	Regular information exchange helps the partnering companies build relationship capital with each other in the form of mutual trust and reciprocal commitment, which in turn leads to better alliance performance
Martin and Emptage (2019)	Knowledge-transfer enablers for successful construction joint ventures	Quantitative	C1; C2; C3	Intent to learn, interaction climate, and articulated goals have a significant positive effect on knowledge acquisition
Pauluzzo and Cagnina (2019)	A passage to India: cultural distrelance issues in IJVs’ knowledge management	Quantitative	C2	The development of knowledge acquisition skills and the relationship of managers and employees increase the willingness to share knowledge
Robson et al. (2019)	Alliance capabilities, interpartner attributes, and performance out-comes in international strategic alliances	Quantitative	C1	Management capability allows firms to build trust, which has an inverted U-shaped relationship with ISA performance
Minbaeva et al. (2018)	Disseminative capacity and knowledge acquisition from foreign partners in international joint ventures	Quantitative	C1; C2	The partnering firm’s codification and articulation ability, willingness to share knowledge, and frequent and effective use of communication channels determine the extent of knowledge acquisition
Nisar et al. (2018)	The entry mode strategy and performance of SMEs: evidence from Norway	Quantitative	C1	Trust and congruity of goals between partner companies have a positive and significant influence on alliance performance
Owens et al. (2018)	Resolving post-formation chall-comenges in shared IJVs: the impostpact of shared IJV structure on inter-partner relationships	Quantitative	C1	Relational conditions (e.g., trust between the partner companies) facilitated the successful management of post-formation challenges
Wong et al. (2018)	Collectivist values for constructive conflict management in international joint venture effectiveness	Quantitative	C3	Collectivist values support cooperative conflict management that in turn facilitates alliance performance. Individualistic values promote competitive conflict management which in turn frustrated alliance performance
Zhang et al. (2018)	Leveraging channel management capability for knowledge transfer in international joint ventures in an emerging market: a moderated mediation model	Quantitative	C2	The mediation effect of channel management capability on alliance performance is particularly salient and becomes stronger when the IJV has a relatively higher ACAP
Tahir and Saba (2017)	Trust-performance relationship in international joint ventures: the moderating roles of structural mechanisms	Quantitative	C1	Trust takes on greater importance in enhancing alliance performance under symmetric dependence and resource complementarity
Vangen (2017)	Culturally diverse collaborations: a focus on communication and shared understanding	Quantitative	C4	Two specific management tensions develop cultural sensitivity and design communication processes. These tension explicate the complexity of culturally diverse contexts that may enhance an alliance ability to yield advantage rather than inertia
Li et al. (2016)	Management of cultural differences under various forms of China-UK higher education strategic alliances	Quantitative	C3; C4	Cultural conflicts occur more frequently in equity joint ventures than in non-equity modes, although the impact of those conflicts is more serious and significant in nons-equity arrangements
Lo et al. (2016)	Relational capital, strategic alliances and learning: in-depth analysis of Chinese-Russian cases in Taiwan	Quantitative	C1	Relational capital between partners, presented through the existence of trust, communication, and openness, has a determinant influence on the effectiveness and quality of learning process in ISA
Park and Vertinsky (2016)	Reverse and conventional knowledge transfers in international joint ventures	Quantitative	C2	The transfer of conventional knowledge presents opportunties for interactions between employees of the IJV and the foreign parent firm, creating bidirectional tacit knowledge flows and a basis for informal mutual learning
Colak et al. (2015)	Some antecedents, moderators and consequences of market orientation in international joint ventures	Conceptual	C1; C4	The concept of market orientation is extended to IJV including the observation of its performance, by offering testable propositions
Gómez-Miranda et al. (2015)	The impact of organizational culture on competitiveness, effectiveness and efficiency in Spanish-Moroccan international joint joint ventures	Quantitative	C4	The level of competitiveness, effectiveness, and/or efficiency of the ISA are influenced by the involvement of staff in management, the degree of centralization of decision taking, and the firms’ emphasis on results or on procedures
Ho and Wang (2015)	Unpacking knowledge transfer and learning paradoxes in international strategic alliances: contextual differences matter	Quantitative	C1; C2	Frequent interactions, strong mutual trust, and reciprocal commitment positively moderate the impact of knowledge protection on ACAP and that of ACAP on alliance performance.-
Isidor et al. (2015)	The impact of structural and attitudinal antecedents on the instability of international joint ventures: the mediating role of asymmetrical changes in Commitment	Quantitative	C1; C2	A model is developed, in which asymmetrical changes in structural antecedents (e.g., partners’ learning) and one attitudinal antecedent (trust) feed forward into IJV instability, mediated by asymmetrical changes in the partners’ commitment
Khan et al. (2015)	Knowledge transfer from international joint ventures to local supliers in a developing economy	Mixed	C1	Formal socialization mechanisms enhance the comprehension and speed of knowledge transfer to local suppliers, informal socialization mechanisms enhance comprehension but not speed
Larimo and Nguyen (2015)	International joint venture strategies and performance in the Baltic States	Quantitative	C1	Differences in the IJV performance depend on parent firms’ objectives, level of trust, and commitment, as well as depending on the performance measures used
Liu et al. (2015)	Fit, misfit, and beyond fit: relational metaphors and semantic fit in international joint ventures	Mixed	C1	Formal ownership moderates the impact of managerial schemas, implied by shared relational metaphors, on the subjective and time-lagged IJV performance
Ott (2015)	Repeated moral hazard in international joint ventures: inter-temporal culturally sensitive incentive schemes for hidden action	Conceptual	C4	Failure of an IJV is determined by the effort levels induced in each lifecycle stage. Besides, managerial efforts and cultural distance influences the stability of IJV.
Varma et al. (2015)	Cultural determinants of alliance management capability – an analysis of Japanese MNCs India	Qualitative	C1	Cultural factors as determinants of alliance management help to establish a managerial blueprint leading to positive alliance outcomes for such ventures
Dadfar et al. (2014)	International strategic alliances in the Iranian pharmaceutical ind ustry: an analysis of key success and failure factors	Mixed	C1; C4	Trust, mutual understanding, and the development of strong inter-organizational relationships are extremely important for the success of ISA.The qualitative study supports the quantitative results and stresses the high importance of the soft aspect of the alliances’ success
Kobernyuk et al. (2014)	International joint ventures in Russia: cultures’ influences on alliance success	Mixed	C4	The organizational culture of an IJV is found to be dependent on the partner company’s culture that dominates and, thus, influences the perceived IJV performance
Liu and Zhang (2014)	Learning process and capability formation in cross-border buyer supplier relationships: a qualitative case study of Taiwanese technological firms	Qualitative	C2	Well-performing ISA integrate inter- and intra-organizational learning, as well as a bidirectional learning processes. A framework of cross-level knowledge flow is proposed with alliance learning antecedents and outcomes
Park and Harris (2014)	Microfoundations for learning within international joint venutres	Qualitative	C2	To build ACAP IJV need appropriate microfoundations at in dividual, process, and structural levels, and coherent interlinkages between them, especially by having IJV managers’ with extensive experience and orientation to learn
Tahir (2014)	The impact of national and organizational cultural differences on international joint venture performance	Conceptual	C1; C4	Partnership trust mediates the relationship between national and organizational cultural differences and IJV performance through a chain of interactive processes
Zheng and Larimo (2014)	Identifying key success factors for international joint ventures in China: A foreign parent perspective from finnish firms	Quantitative	C1; C4	Regarding alliance performance at IJV operation stage, partner commitment is the key success factor
Likhi and Sushil (2013)	Building international strategic alliance capability: a case research-based insights	Quantitative	C1; C4	The external context, leadership-support, and commitment are key determinants for alliance performance
Mohedano-Suanes and Benavides Espinosa (2013)	Technology transfer, specific local knowledge and entrepreneurial partner control in international joint ventures	Quantitative	C1; C4	During the post-formation stage, the configuration of entrepreneurial partner control over the IJV is conditioned by both technology transfer and by learning specific local knowledge. Moreover, the negative relation between trust and control, suggested by the literature, is limited to cases where trust has broken down
Pajunen and Fang (2013)	Dialectical tensions and path dependence in international joint venture evolution and termination	Quantitative	C3	This study contributes to research by explaining the path dependent nature of dialectical tensions in the evolution and survival of IJV
Wai On et al. (2013)	Top management team trust, behavioural integration and the performance of international joint ventures	Mixed	C1; C4	Trust mediates the relationship between the behavioral integration of top managers in Sino-foreign IJV and its perfomance. The effects of business similarity and partner national cultural distance on IJV performance were not mediated by trust
Yitmen (2013)	Organizational cultural intelligence: a competitive capabilityfor strategic alliances in the international construction industry	Quantitative	C4	The partnering firms’ cultural intelligence works as a cros
Damanpour et al. (2012)	Organizational culture and partner interaction in the management interaction in the management of international joint ventures in India.-	Quantitative	C1; C4	Organizational cultural differences negatively affect IJV performance through the mediation of partner interaction processes
Li et al. (2012)	Symbiotic ownership, cultural alignment, and firm performance: a test among ISA	Quantitative	C4	ISA benefit from building a collectivistic organizational culture, when they are highly interleaved with the industry
Nielsen and Gudergan (2012)	Exploration and exploitation fit and performance in international strategic alliances	Mixed	C2; C4	Regarding upstream innovative performance, prior experience with the partner is potentially damaging for this type of performance, and trust and cultural distance do not play significant roles. Regarding downstream market performance, theses factors are beneficial
Park et al. (2012)	Korean international joint ventures: how the exchange climate affects tacit knowledge transfer from foreign parents	Quantitative	C1; C2; C3	Conflict resolution and cooperation positively affect tacit knowledge transfer, but communication does not. Cultural distance does not influence tacit knowledge acquisition. although tacit knowledge acquisition positively influences IJV performance
Tey and Quah (2012)	Effect of inter partner fit in international joint venture knowledge transfer	Conceptual	C4	Inter-partner fit is important regarding the IJV knowledge transfer which is highlighted by proposing a conceptual model
Adnan et al. (2011)	Success criteria for international joint ventures: the experience of Malaysian contractors in the Middle East	Quantitative	C1; C2	Critical factors in IJV partner selection are related to interpartner trust, experience, personal knowledge of the partner companies, commitment, and human resources management
Baughn et al. (2011)	Social capital and human resource management in international joint ventures in Vietnam: a perspective from a transitional economy	Quantitative	C1; C2	IJV performance is predicted by training and by the level of trust and cooperation between foreign and local personnel
Farrell et al. (2011)	Antecedents and performance consequences of learning success in international joint ventures	Quantitative	C1; C2; C3	Partner companies with higher levels of learning orientation/success contribute more to alliance performance. Partner companies that are successful in achieving their learning goals, and improve their knowledge, will be winners in terms of improving their own business performance alike
Kim et al. (2011)	Resources and performance of international joint ventures: the moderating role of ACAP	Quantitative	C2	Performance of IJV seems to be driven by the complementary resources of partner companies in combination with ACAP of IJV
Lee et al. (2011)	Parent control mechanisms, knowledge attributes, knowledge acquisition and performance of IJV in Taiwan service industries	Quantitative	C2	The partner companies require a "personnel training" control mechanism as a guide for gaining codified knowledge from the other partner companies
Bener and Glaister (2010)	Determinants of performance in international joint ventures	Quantitative	C1; C4	Alliance performance positively relates to trust between the partner companies. The negative relationship between alliance performance and and national cultural differences was not supported. The negative relationship to corporate cultural differences was partially supported
Choi et al. (2010)	Communication, utilization, and performance in international strategic alliances: an investigation of the post-formation process	Quantitative	C1	Utilization of resources is found to fully mediate the relationships between the three significant communication factors (formal, two-way, participative) and alliance performance
Fang and Zou (2010)	The effects of absorptive and joint learning on the instability of international joint ventures in emerging economies	Quantitative	C2	Local and foreign IJV parties’ absorptive learning capacity decreases one party’s dependence on the other, while joint learning capacity in IJV increases both parties’ dependence on each other
Girmscheid and Brockmann (2010)	Inter- and intraorganizational trust in international construction joint ventures	Mixed	C1	Trust is a mechanism that allows to reach goals efficiently within the setting of IJV
Hsieh et al. (2010)	Risk perception and post-formation governance in international joint ventures in Taiwan: the perspective of the foreign partner	Quantitative	C3; C4	Conflicts between partners, opportunistic behavior by the local partner, cultural differences, and perceived partner misfit are related to foreign partners’ risk perceptions.
Park and Son (2010)	What matters to managerial knowledge acquisition in international joint ventures? High knowledge acquirers versus low knowledge acquirers	Quantitative	C1; C2; C4	Trust between partner companies, international experience of IJV employees, and foreign parent’s support in various managerial functions will considerably increase the extent of knowledge acquisition for IJV
Yan et al. (2010)	An exploration of managerial discretion and its impact on firm performance	Quantitative	C1	Contractual control does not engender a statistically significant relationship between greater managerial discretion and superior alliance performance
Chen et al. (2009)	The influence of partner characteristics and relationship capital on the performance of international strategic alliances	Quantitative	C1	Partner characteristics indirectly affect alliance performance through relationship capital. Moreover, mutual trust and information sharing affect alliance performance through reciprocal commitment
Drouin et al. (2009)	Investigation of contextual factors in shaping HR approaches and determining the success of international joint venture projects: evidence from the Canadian telecom industry	Mixed	C1; C4	The HR approaches in the IJV formation stage, relating to its performance, is influenced by key individual, organizationail, and and cultural factors such as composition of the IJV team, company’s distribution of power and decision-making style, organizational frame of reference, and socio-cultural distance between IJV partners
Luo (2009)	From gain-sharing to gain-generation: the quest for distributive justice in international joint Ventures	Quantitative	C1	Distributive justice is an important variable affecting IJV stability via the mediation of inter-organizational attachment
Pak et al. (2009)	Understanding IJV performance in a learning and conflict mediated context	Quantitative	C2; C3; C4	Cultural similarity had a positive effect on cross-border learning, which was reinforced by learning capacity, and which in turn led to better alliance performance. Conflicting relations between partner companies and heterogeneous cultural backgrounds were shown to discourage learning
Park et al. (2009)	Acquisition of managerial knowledge from foreign parents: evidence from Korean joint ventures	Quantitative	C1; C2; C4	The IJV intent to learn, international experience, level of trust between partner companies, business relatedness, and active managerial engagement of the partner companies are the most important factors explaining the level of knowledge acquisition within the IJV
Wilson and Brennan (2009)	Relational factors in UK-Chinese international joint ventures	Qualitative	C1	Trust appears to be the most important of the relational factors on IJV performance, followed by commitment
Avny and Anderson (2008)	Organisational culture, national culture and performance in international joint ventures based in Israel	Qualitative	C1; C4	Neither organizational nor national culture had much impact on alliance performance. However, trust seems to play an important mediating role
Fang et al. (2008)	Trust at different organizational levels	Qualitative	C1	Inter-organizational and agency trust motivate resource investments in the
Farrell et al. (2008)	Market orientation, learning orientation and organisational performance in IJV	Quantitative	C2	For IJV, a market orientation has a more positive impact on its performance than a learning orientation
Kwon (2008)	Antecedents and consequences of international joint venture partnerships: a social exchange perspective	Qualitative	C1; C4	The competitive relationship between partners was shown not to exert a significant negative influence on both the trust-commitment partnerships and the effectiveness of IJV
Lin and Wang (2008)	Enforcement and performance: the role of ownership, legalism and trust in international joint ventures	Quantitative	C1	Trust is found to be positively associated with alliance performance across all samples. However, only for Western partner companies, legalism has a significant impact on alliance performance and moderates the effect of trust
Ozorhon et al. (2008)	Implications of culture in the performance of international construction joint ventures	Quantitative	C4	Differences in the organizational culture have a greater impact on IJV performance than differences in national culture
Robson et al. (2008)	Drivers and performance outcomesa of trust in international strategic alliances: the role of organizational complexity	Qualitative	C1	While inter-partner trust is positively associated with alliance performance, this relationship becomes stronger when alliance size declines
Steensma et al. (2008)	The evolution and internalization of international joint ventures in a transitioning economy	Quantitative	C2; C3	Extensive knowledge transfer to an IJV in a transitioning economy combined with high levels of conflict increases the likelihood of the foreign partner gaining full ownership When there is extensive knowledge transfer and low conflict, the local parent is more likely to internalize the IJV
Zhan and Luo (2008)	Performance implications of capability exploitation and upgrading in international joint ventures	Quantitative	C1: C2	IJV in a foreign emerging market tend to perform better when they possess greater abilities to exploit current resources contributed by foreign and local partners and when they continuously upgrade and develop new capabilities
Demir and Söderman (2007)	Skills and complexity in management of IJVs: exploring Swedish managers’ experiences in China	Mixed	C4	One can evaluate learning within an ISA as a race by both partner companies to learn from and eventually outperform the other party
Lin (2007)	Appointing a general manager in Sino-US joint ventures: partner competence and organizational consequences	Quantitative	C4	When the general manager is Chinese rather than American there is conflict on daily personnel management issues and the overall levels of partner satisfaction and relationship commitment decreases
Lyles and Salk (2007)	Knowledge acquisition from foreign parents in international joint ventures: an empirical examination in the Hungarian Context	Quantitative	C2; C3	The relationship between knowledge acquisition and alliance performance is significant. Knowledge acquisition from the foreign partner, which enhance IJV knowledge acquisition, affect some dimensions of IJV performance more than others
Mohr and Puck (2007)	Role conflict, general manager job satisfaction and stress and the performance of IJVs	Qualitative	C3	Managers experiencing a high level of role conflict also report lower job satisfaction and higher job stress. High job stress is related low IJV performance, while there is no statistically significant relationship between job satisfaction and IJV performance
Ng et al. (2007)	The effect of trust on international joint venture performance in China	Quantitative	C1	Trust influences IJV performance and the moderating effect of trust on the relationship between IJV performance and local reliance/experience of executives were confirmed for the senior executive sample
Nielsen (2007)	Determining international strategic alliance performance: a multi-dimensional approach	Quantitative	C1; C4	There is a significant relationship between alliance performance and host country risks well as partner reputation. During the operation of the ISA, trust and cultural distance are essential
Watts and Hamilton III (2007)	Excessive resource control and strategic alliance failure	Conceptual	C1; C4	The combination of resource dependency theory with trust literature helps to explain how antecedent conditions and resource control can increase the risk of failure in ISA
Thuc Anh et al. (2006)	Knowledge acquisition from foreign parents in international joint ventures: an empirical study in Vietnam	Quantitative	C2; C4	ACAP contributes to the level of knowledge acquisition reported by IJV. Moreover, critical factors predicting knowledge acquisition are investment in training, employees’ ability to learn, and joint participation
Brouthers and Bamossy (2006)	Post-formation processes in eastern and western European joint ventures	Quantitative	C1; C4	IJV performance is dependent on the post-formation processes to overcome barriers to success created by differences in natural culture and trust
Mehta et al. (2006)	Strategic alliances in international distribution channels	Quantitative	C1; C2	Similarities for fostering cooperation in international distribution alliances were detected, but on a cross-national basis disparate emphases on management practices were observed when regarding its relation to alliance performance
Robson et al. (2006)	Anxiety of dependency in international joint ventures? An empirical study of drivers and consequences of relationship insecurity	Quantitative	C1	The partner companies dependence on each other negatively affect insecurity. Thus, insecurity not only reduces IJV performance directly, but also lowers the quality of inter-partner communication, which in turn dampens IJV performance
Gong et al. (2005)	Human resources and international joint venture performance: a system perspective	Quantitative	C4	IJV encompass two distinct but inter-related human resource sets: a set within the venture, having a detrimental impact on its performance; a set associated mainly with the relational tension along the partners and the venture subsystem
Kandemir and Hult (2005)	A conceptualization of an organizational learning culture in international joint ventures	Conceptual	C2; C4	IJV achieve superior performance by higher levels of innovativeness (openness to new ideas) and innovation capacity (capacity to implement innovations), which are associated with its organizational learning culture
Mohr and Puck (2005)	Managing functional diversity to improve the performance of international joint ventures	Mixed	C1	Although functional diversity has a negative impact on IJV performance, managers can influence the magnitude of this impact through trust, commitment, and communication which function as moderating variables
Robson and Katsikeas (2005)	International strategic alliance relationships within the foreign investment decision process	Mixed	C4	Top management attitude towards an ISA is negatively associated with scope of cooperation analysis, but only where collaborative history exists. Scope of cooperation analysis positively influences willingness to invest
Styles and Hersch (2005)	Relationship formation in international joint ventures: insights from Australian-Malaysian international joint ventures	Qualitative	C1	During the five stages of formation four dimensions of trust and three dimensions of commitment play prominent roles. which should be focused on to improve chances of success
Thuy and Quang (2005)	Relational capital and performance of international joint ventures in Vietnam	Qualitative	C1; C3	Investment in relational capital between partner companies is critical for the success of the IJV. Moreover, the mediating roles of inter-partner flexibility, goal clarity, and conflict management have to be stressed
Dhanaraj et al. (2004)	Managing tacit and explicit knowledge transfer in IJVs: the role of relational embeddedness and the impact on performance	Qualitative	C1; C2	Trust and shared values play an important role in the transfer of tacit knowledge. The influence of transferred tacit knowledge on IJV performance stems principally from its indirect effect on the learning of explicit knowledge
Kauser and Shaw (2004)	The influence of behavioural and organisational characteristics on the success of international strategic alliances	Quantitative	C1; C3	Behavioral characteristics play a more significant role in explaining overall alliance performance compared to organizational characteristics. For instance, commitment, trust, and communication are found to be good predictors of success. Conflict hampers alliance performance
Tsang et al. (2004)	Knowledge acquisition and performance of of international joint ventures in the transition economy of Vietnam	Quantitative	C1; C2; C3	Certain IJV characteristics influence the joint venture’s knowledge acquisition from its partner companies and the amount of knowledge acquired affects IJV performance. Parental conflict, commitment, and receptivity affect knowledge acquisition
Beamish and Berdrow (2003)	Learning from IJVs: The unintended outcome	Quantitative	C2	Production-based IJV are not motivated by learning, as no direct relation to IJV performance is found
Boersma et al. (2003)	Trust in international joint venture relationships	Mixed	C1	In the early stages of an IJV, promissory-based trust predominates, and as the IJV progresses, competences-based trust emerges. Goodwill-based trust is always important
Child and Yan (2003)	Predicting the performance of international joint ventures: an investigation in China	Quantitative	C2	The main performance predictors are the partner companies experience with international business/IJV and the quality of resources they provide to the IJV
Demirbag et al. (2003)	Trust, inter-partner conflicts, cultural distance, commitment and joint venture performance: an empirical analysis of international joint ventures in Turkey	Quantitative	C1; C3; C4	Trust and forbearance are key dimensions for understanding the performance of IJV and ISA alike
Vaidya and Nasif (2002)	Learning effectiveness in international joint ventures (IJVs): a conceptual framework	Conceptual	C1; C4	The relationship between learning effectiveness, as alliance performance, and several variables (e.g., trust, commitment, organizational culture, and national cultural similarity) is assumed
Zeybek et al. (2003)	Perceived cultural congruence’s influence on employed communication strategies and resultant performance: a transitional economy international joint venture illustration	Quantitative	C1; C4	The more culturally congruent a company perceives its IJV partner to be, its communications employed become less frequent and more influential in terms of content. Results indicate that the more frequent and more formalized communication strategies employed by a partner company are, the greater IJV partner’s self-reported IJV performance
Currall and Inkpen (2002)	A multilevel approach to trust in joint ventures	Conceptual	C1	IJV trust, in particular conceptualized and measured at the individual, group, and organizational level, is explained, as it is essential for IJV success
Fryxell et al. (2002)	After the ink dries: the interaction of trust and control in US based international joint ventures	Quantitative	C1	Social control mechanisms and perceptions of IJV performance positively relate to each other, but only in the presence of affect-based trust between the partner companies
Iles and Yolles (2002)	International joint ventures, HRM and viable knowledge migration	Conceptual	C2	A model of viable knowledge development in IJV and its relationship to HRM, involving knowledge migration, appreciation, and action is proposed which leads to organizational learning
Li et al. (2002)	Multi-cultural leadership teams and organizational identification in international joint ventures	Mixed	C1; C3	Organizational identity and identification can be a valuable tool to facilitate the understanding of top management team functioning and IJV performance
Luo (2002)	Building trust in cross-cultural collaborations: toward a contingency perspective	Quantitative	C1; C4	Trust plays a stronger role in improving ISA performance when commitment to the ongoing partnership is higher Cultural distance between alliance parties does not moderate the trust-performance link
Pothukuchi et al. (2002)	National and organizational culture differences and international joint venture performance	Quantitative	C4	The negative effect from culture distance on IJV performance originates more from differences in organizational culture than from differences in national culture
Gong et al. (2001)	Role conflict and ambiguity of CEOs in international joint ventures: a transaction cost perspective	Quantitative	C1; C3	Role conflict is lower when the foreign party is dominant in the venture, but higher when the local party is dominant. Role conflict and ambiguity are inversely related to cultural distance. Neither variable has an effect on performance
Griffith et al. (2001)	Knowledge transfer as a means for relationship development: a Kazakhstan-foreign international joint venture illustration	Quantitative	C1; C2	IJV with higher levels of knowledge transfer result in higher levels of partner companys’ commitment to and satisfaction with their relationships, thus supporting a relationship development perspective of knowledge transfer
Hambrick et al. (2001)	Compositional gaps and downward spirals in international Joint venture management groups	Conceptual	C1	Compositional gaps in IJV management teams will accentuate distinct managerial coalitions. A model is proposed that has implications for IJV performance
Kauser and Shaw (2001)	International strategic alliances: the impact of behavioral charcteristics on success	Quantitative	C1; C3	High levels of coordination, commitment, trust, and communication are good predictors of ISA success. While conflit is present in ISA, it is found to hinder performance
Lane et al. (2001)	Absorptive capacity, learning, and performance in international joint ventures	Quantitative	C1; C2	Unexpectedly, trust and management support from the foreign partner company are associated with IJV performance, but not learning
Salk and Shenkar (2001)	Social identities in an international joint venture: an exploratory case study	Qualitative	C1	Social identity enactments by team members mediate the relationship of contextual variables, both environmental and structural, with group and organizational outcomes
Schuler (2001)	Human resource issues and activities in international joint ventures	Conceptual	C2	A four-stage model of IJV, based on the importance of learning and long-term relationships, is used to generate several propositions regarding HR issues in IJV
Cullen et al. (2000)	Success through commitment and trust: the soft side of strategic alliance management	Mixed	C1	A dynamic model of trust and commitment is developed which shows how the dynamics of trust and commitment affect the performance of ISA
Demirbag and Mirza (2000)	Factors affecting international joint venture success: an empirical analysis of foreign-local partner relationships and performance in joint ventures in Turkey	Quantitative	C1; C3	A dynamic model of inter-partner relations and IJV performance is developed which indicates the causal connections between conflict, commitment, control and IJV performance
Jennings et al. (2000)	Determinants of trust in global strategic alliances: Amrad and the australian biomedical industry	Quantitative	C1	A process model of trust is developed which describes how trust can be created and expanded between ISA partner companies, including implications for alliance performance
Steensma and Lyles (2000)	Explaining IJV survival in a transitional economy through social exchange and knowledge based perspectives	Quantitative	C3	An imbalance in the management control structure between the partner companies leads to parental conflict and an increased likelihood of IJV failure
Fey and Beamish (1999)	Strategies for managing Russian international joint venture conflict	Quantitative	C3	Nine strategies for managing intra-IJV conflicts are presented, such that it will have a minimal negative impact on IJV performance
Li et al. (1999)	Building effective international joint venture leadership teams in China	Qualitative	C4	Five key elements of IJV-related leadership issues (team composition, processes, structure, incentives, and the leader’s behavior) are identified that have important implications for IJV success
Lin and Germain (1999)	Predicting international joint venture interaction frequency in U.S.-Chinese ventures	Quantitative	C1; C4	Relationship commitment positively predicts IJV interaction frequency, which is understood as an indicator of performance, and cross-cultural adaptation
Si and Bruton (1999)	Knowledge transfer in international joint ventures in transitional economies: the China experience	Qualitative	C2	Satisfaction with the performance of many IJV in China is declining, which can be traced in part to the inability of many companies to assess their knowledge acquisition goals within the IJV
Ariño and de la Torre (1998)	Learning from failure: towards an evolutionary model of collaborative ventures	Qualitative	C1; C2	Positive feedback loops are critical in evolutionary processes, relationship quality is both an outcome and a mediating variable and procedural issues are critical from the start to build mutual trust
Inkpen (1998)	Learning and knowledge acquisition through international strategic alliances	Conceptual	C2	In bringing together companies with different skills, know-ledge bases, and organizational cultures, ISA create unique learning opportunities for all partnering companies
Inkpen and Dinur (1998)	Knowledge management processes and international joint ventures	Qualitative	C2	Possibilities are portrayed, how IJV can be integrated into a company’s dynamic system of knowledge creation, to strengthen its knowledge transfer
Lin and Germain (1998)	Sustaining satisfactory joint venture relationships: the role of conflict resolution strategy	Quantitative	C3; C4	A conceptual framework is presented that links IJV context (e.g., cultural similarity) and the partnering companies conflict resolution strategies to IJV performance
Ramaseshan and Loo (1998)	Factors affecting a partner’s per-ceived effectiveness of strategic business alliance: some Singaporean evidence	Quantitative	C1; C3	The factors commitment to alliance, inter-organizational communication, and inter-organizational trust are positively related to alliance performance. Dysfunctional conflicts are found to have a negative impact
Barkema and Vermeulen (1997)	What differences in the cultural backgrounds of partners are detrimental for international joint ventures?	Quantitative	C4	The impact of cultural distance on IJV survival does not disappear over time
Cyr and Schneider (1996)	Implications for learning: human resource management in east west joint ventures	Qualitative	C2; C4	Encouraging employee performance, satisfaction, and learning in IJV in transition economies will lead to organizational success
Eroglu and Yavas (1996)	Determinants of satisfaction with partnership in international joint ventures: a channels perspective	Quantitative	C1; C3	Perceived partner company contribution is the most powerful predictor of IJV performance and the ability to manage and resolve conflicts becomes a critical requirement
Lyles and Salk (1996)	Knowledge acquisition from foreign parents in international joint ventures: an empirical examination in the Hungarian context	Quantitative	C2; C3; C4	Learning from the foreign partner company is thought to be critical. Adaptation mechanisms, such as capacity to learn, articulated goals, and structural mechanisms (e.g. training, managerial assistance), were positively associated with knowledge acquisition
Geringer and Frayne (1993)	Self-efficacy, outcome expectancy and performance of international joint venture general managers	Mixed	C4	Self-efficacy and outcome expectancy of IJV general managers significantly relate to IJV performance
Turpin (1993)	Strategic alliances with Japanese firms: myths and realities	Qualitative	C1	As several examples in business practice show, IJV in Japan can work when the partner companies avoid complexities, trust each other and commit themselves to the new venture
Hamel (1991)	Competition for competence and inter-partner learning within international strategic alliances	Qualitative	C1; C2	Not all partner companies are equally adept at learning. Asymmetries in learning alter the relative bargaining power of these parties and they may have competitive as well as collaborative aims

During the result section (in particular chapter 6.2–6.4) we only used the term “alliance performance” when referring to the *various ways of expressing alliance performance*. This was done to minimize the complexity in presenting the identified influencing factors. However, a discussion of the different performance constructs, with regard to the factors of influence, shall be not omitted. Hence, this is made subsequently by steadily referring to the derived categories.

The category “relational factors” predominantly encompasses “organizational performance” constructs. Especially scales measuring “management satisfaction” (e.g.,Kwon 2008; Nisar et al. 2018) as well as “knowledge acquisition” (e.g., Tsang et al. 2004; Martin and Emptage 2019) function as common ways to capture alliance performance when considering this category. For instance, those measures were used to analyze the factor trust on the individual (e.g., Robson et al. 2019) and organizational level (e.g., Lin and Wang 2008). “Operational performance” constructs are represented much less in this category, although team commitment (e.g., Owens et al. 2018) as well as inter-partner trust (e.g., Mohedano-Suanes and del Mar Benavides-Espinosa 2013) relate to measures like “(in)stability” or “survival” of an ISA. As explained in the result section, “financial performance” constructs are rarely used in behavioral ISA-related studies in general. Solely articles dealing with trust (e.g., Owens et al. 2018) included this possibility to analyze alliance performance.

In the category “learning and knowledge” almost only “organizational performance” constructs are used – usually this is “knowledge acquisition” (e.g., Park and Vertinsky 2016; Martin and Emptage 2019). ACAP, as an influencing factor on the organizational level, is also often related to “management satisfaction” (e.g., Lyles and Salk 2007; Zhang et al. 2018), but also relates to “operational performance” constructs like “(in)stability” (e.g., Fang and Zou 2010). “Financial performance” constructs are not present in this category at all.

“Organizational performance” constructs also dominate in the category “conflict”. In particular, scales that refer to “management satisfaction” are found (e.g., Kauser and Shaw 2004; Mohr and Puck 2007). This is understandable, since occupational conflicts and satisfaction are constructs that are mutually dependent, wherefore their relationship is often investigated empirically (e.g., Simães et al. 2021). Nevertheless, inter-organizational conflicts relate to “operational performance” constructs like “(in)stability” as well (e.g., Hsieh et al. 2010). The category “conflict” also encompasses no “financial performance” constructs.

The last category “other (unrelated) factors” also encompasses numerous articles that make use of “organizational performance” constructs (e.g., Park et al. 2009). It is noticeable that “knowledge acquisition” is primarily used as alliance performance when relating to expatriate issues/management as an influencing factor (e.g., Lyles and Salk 1996). This makes sense, since one of the main objectives for sending an employee (to an ISA) abroad is knowledge transfer (Cheong et al. 2019). Thus, the use of “knowledge acquisition” as a performance construct is meaningful, in order to quickly verify, if and how the expatriate has acquired knowledge with or from the partner company. With regard to national and organizational cultural differences, the picture is divided. On the one hand, cultural differences relate to “organizational performance” constructs (e.g., Pak et al. 2009; Gómez-Miranda et al. 2015) like “management satisfaction”, but on the other hand also to “operational performance” constructs (e.g., Hsieh et al. 2010; Ott 2015) like “(in)stability” or “survival”.

## Future research

Starting with implications for future research, Fig. 4 functions as a guiding structure for an explication of which topics in each behavioral level would be most fruitful for upcoming research to pursue. The figure highlights needs for future research in terms of currently ambiguous results and topics of scarce research in general that demand additional empirical validation.

Starting with the *individual level*, researchers who have addressed the influence of role conflicts have not agreed. The fact that at least three parties, the ISA and two partnering companies, direct individuals like managers can cause role conflicts, since different roles may need to be occupied, in order to satisfy these stakeholders. However, whether such role conflicts have positive or negative impacts on alliance performance is not clear, as some academics argued that they can increase motivation (e.g., Gong et al. 2001), while others have found that they lead to occupational stress, harming alliance performance (e.g., Li et al. 2002; Mohr and Puck 2007). Therefore, additional examination of the relationship between role conflicts and the performance of ISA is needed. Such a validation could be done by considering additional quantitative studies that, for instance, follow experimental approaches and include supplementary mediating or moderating variables (e.g., organizational commitment). In fact, occupational stress could be another topic for future research, as the connection of this factor to alliance performance was observed only by Mohr and Puck (2007), although this variable is often linked with role conflicts and an important influencing factor in ordinary organizational behavior research (e.g., Kariv 2008).

The *group-related level* is the least represented level, as only 30 articles have been identified. Hence, there is significant potential for further academic advancement on this level (e.g., composition of and commitment in teams). Especially research on leadership topics is scarce, as despite the view that leadership is essential for ISA and their performance (e.g., Li et al. 1999; Likhi and Sushil 2013), no explicit research on such factors is extant in our sample. However, studies that have been situated in the ordinary realm of organizational behavior have addressed aspects of leadership like the style of leadership (e.g., Idris and Mohd Ali 2008; Mgeni 2015) and perceptions of supervisors’ behavior (e.g., Jing and Avery 2008; Steyrer et al. 2008) when analyzing (business) performance and stressed their importance. Moreover, the fact that ISA mirror cross-border cooperations, which encompasses at least two legally distinct organizations that are situated in different countries (Gulati 1998; Nielsen and Gudergan 2012), implies that these leaders are responsible for employees that date from disparate organizational as well as national cultural backgrounds. Thus, the behavior of leaders and their interaction with certain individuals within ISA are of special importance and have to be deeply analyzed in future research. Beyond that, our analysis shows no empirical articles that have dealt with learning processes in (management) teams. However, researchers observed that learning takes place on the group-related level of ISA as well (Inkpen and Dinur 1998; Schuler 2001), wherefore we suggest that future research should add corresponding variables to behavioral studies that deal with alliance performance. Altogether, exploratory research methods like interview studies seem to be appropriate on this level. Qualitative data can help to understand novel topics more deeply (Mey and Mruck 2020) and are often the starting point when considering new topics of research.

The *organizational level* has been the most extensively analyzed, although some topics demand additional research to clarify contradictory results or to strengthen existing findings. For instance, various academics have agreed that knowledge acquisition between the partnering companies in an ISA amplifies alliance performance (Pak et al. 2009; Liu and Zhang 2014), while others have claimed that such processes diminish the chances of survival (Mohedano-Suanes and del Mar Benavides-Espinosa 2013). In general, the literature has made clear that a reciprocal acquisition of knowledge can lead to a “learning race” between partnering companies (Fang and Zou 2010), which may cause instability, as the absorption of knowledge can eliminate former dependencies between partners. Therefore, knowledge acquisition should be further analyzed to clarify whether “absorptive learning” or “joint learning” is more or less auxiliary for alliance performance. Another relationship that remains to be straightened out is that of inter-organizational conflicts. Some authors stress a negative (e.g., Hsieh et al. 2010) and others a positive influence (e.g., Demirbag et al. 2003) on alliance performance, which leaves a contemporary confusion. Finally, future research should continue to deal with the topic of cultural differences. By viewing national cultural differences, one recognizes that the work of Hofstede (1983) has often been used as a basis for empirical research. However, literature has revealed weaknesses in this approach, so future research could use other conceptualizations (e.g., GLOBE study, House et al. 2004) as a foundation to enforce novel studies. Moreover, contrary to our results a recent literature review, that dealt with culture in international management, stated a positive relationship between national cultural distance and (alliance) performance, leading to a further inconsistency that needs to be clarified (Srivastava et al. 2020). In addition, since organizational cultural differences have been diversely conceptualized in research, academics should agree on specific conceptualizations to use, when measuring this factor, so results can be comparable. Overall, further studies on the organizational level should primarily conduct quantitative studies, as the already gained findings need to be validated, so that contradictory results will be enlightened.

Coming to the *multidimensionality* of factors that affect alliance performance, future research could focus on factors that reside in two or even all three behavioral levels at once and, thus, belong to one of the derived categories. While some articles have already done so, they are scarce. For instance, only two studies in our sample analyzed the multidimensionality of trust (Currall and Inkpen 2002; Girmscheid and Brockmann 2010), only one study observed the dialectical tensions that can take place across all three levels (Pajunen and Fang 2013), and only one conceptual study focused the multidimensionality of learning (Schuler 2001). Based on these insights, we suggest that a debate on the pluralistic understanding of specific variables is worthwhile, as research has not comprehensively compared their influences (e.g., trust, commitment, conflicts, learning). It would be helpful to determine, which manifestation of a variable within a certain category might be more or less influential (e.g., personal trust vs. inter-partner trust). Therefore, we claim that future research should undertake comparative analyses of variables that cross more than one behavioral level to determine, which manifestation might have the highest influence on the performance of ISA.

Regarding alliance performance itself, our result section and discussion highlight that three constructs have been applied by academics to uncover the influence of behavioral factors: financial, operational, and organizational performance – as assumed in the fundamental performance literature (e.g., Ariño 2003). Moreover, by analyzing our literature sample we came across various sub-constructs of alliance performance that are linked to these three pivotal constructs (e.g., (in)stability, management satisfaction). The question becomes distinct, if and how these sub-dimensions, with regard to their operationalization/measurement, relate to each other. For instance, a chain of causation for several variables is close to hand (e.g., knowledge transfer and organizational learning), since some of these might be rather functioning as a mediator than an independent variable and, hence, just have an indirect effect on performance outcomes than being the unblemished performance variable one should consider in upcoming studies. Additionally, it would be of interest to consider more than one performance construct in behavioral studies to understand possible reciprocal relationships even further. This could be particularly done by following quantitative research strategies.

Fundamentally, we suggest that behavioral variables should be included in future research more often as *moderating or mediating variables*. Doing so will help to clarify the empirical relationships that have already been analyzed and offer the possibility of extending theoretical models that are related to this research domain. Following quantitative methods even further, research could think of applying subsequent *meta-analytical approaches* to validate our qualitative findings of this systematic literature review. Such a study could be undertaken for the whole body of literature, for one of the four categories we have built (e.g., relational factors) or only for specific variables (e.g., trust). Furthermore, several quantitative studies in our literature sample do not apply research methods that allow *conclusions regarding causality* (e.g.,Mohr and Puck 2007; Choi et al. 2010). The application of experimental methodologies (Aguinis and Bradley 2014) or longitudinal studies (Kling et al. 2017) could lead to conclusions regarding causality of specific variables with regard to their influence on alliance performance. This should especially be done for variables that show ambiguous results (e.g., knowledge acquisition, inter-organizational conflicts). Compared to the organizational level, few articles relate to the individual and especially to the group-related level, so several factors of influence are just beginning to be analyzed empirically. *Explorative approaches* are likely to be fruitful in enriching certain topics like role conflicts and leadership. This advancement can be assumed since qualitative research has the power to understand complex phenomena that have become apparent in (business) practice and are new to academic research. Moreover, such research approaches allow to build or advance theory which can be a groundwork for further (quantitative) studies (Mey and Mruck 2020). Finally, the research domain considered here should be included in *journals* that predominantly treat issues *of organizational behavior*. This could reveal novel insights and push research of ISA and their performance in promising directions, as only academics in strategic/international management dealt with this topic so far. Thus, this literature field could benefit from applying theoretical or methodological approaches that are grounded in behavioral research.

## Implications and concluding remarks

Overall, we carefully described the search strategy executed, along with the inclusion and exclusion criteria, to ensure objectivity and reliability. In addition, the created decision tree (see Appendix 1) served as a guide in analyzing the databases and articles’ content, which increased transparency and traceability of our review. Nevertheless, the review faces certain limitations.

First, as we focus on research and its development, *practice-oriented journals* were excluded. Moreover, only publications in *English language* were included to address a wide international audience. However, these choices could have led to a population of articles that does not represent every piece of literature that could have been relevant to our research questions.

Second, the process we used to select articles, particularly the creation of our *search string*, was subjective in nature. This subjectivity results especially from the third part of our string on behavioral constructs, as it is based on textbooks. By regarding the magnitude of themes linked to organizational behavior, other researchers might have included other terms, as different academics tend to have different research focuses. Therefore, the decisions we made in creating the search string could have biased our numerical results (see Figs. 2 and 3) and our in-depth content analysis, as these decisions laid the foundation for all following methodological steps.

Third, we chose a *database-driven approach* to prevent omitting relevant articles. However, this approach may have missed in-press articles and, thus, limited the coverage of the literature (Hiebl 2021). Furthermore, the approach we used initially screened only title, abstract and keywords when we analyzed the determined databases (EBSCO, Web of Science, and Scopus). A full-article-length screening of all hits concerning these databases could have achieved a larger sample. One also has to criticize the quality of our literature sample, which is a consequence of using the database-driven approach. We just tracked the latest published impact factors regarding our literature sample and formed an average merit. However, this kind of measure is not widely accepted in the literature, as its creation is controversial (Hiebl 2021). Therefore, we additionally created a figure that shows the journals most often cited linked to the articles of our literature sample (see Fig. 3), to increase transparency. Even so, the sample is not as high-quality as it might have been, when we had analyzed selected academic journals.

As a result of our review, some implications for practitioners became apparent and particularly three aspects have to be named. Firstly, a trustful relationship between the partnering companies is essential – especially when starting a collaboration to guarantee its long-term success (Cullen et al. 2000). Thus, the partner companies must be able to ensure for themselves that they can trust the other part and accept vulnerability which is required when successfully creating an ISA (Luo 2002). Secondly, due to the overarching research context of international management, the consideration of national cultural issues is essential. Since ISA always include at least two partnering companies that are settled in different countries (Nielsen and Gudergan 2012), conflicting situation may occur, because those cultures could be fundamentally different. Managers of ISA have to keep this in mind, when selecting a partner company for their alliance project. Thirdly, our insights might help managers to implement or improve their conflict management. We stressed that three conflict types can take place in an ISA (role conflicts, inter-personal conflicts, and inter-organizational conflicts). This trichotomy could be new to some practitioners, although knowing it may help managers of ISA to understand different situations of conflict better and handle them successfully.

All in all, this comprehensive and systematic review of the literature sheds light on the effects of behavioral factors on ISA (and their performance), for what research claimed to analyze (e.g., Nippa and Reuer 2019; Srivastava et al. 2020), since ISA are a prominent mode of international venture cooperation that aspire innovation and competitiveness (Nielsen and Nielsen 2009; Parmigiani and Rivera-Santos 2011; Haase and Franco 2015).

The answers to our research questions (e.g., increasing publications, multidimensionality of certain influencing factors) strengthen the body of literature. We also identified research gaps in terms of ambiguous results or scarce literature regarding specific factors and hope that this review will help future researchers to improve the quality of their research embedded in this academic realm.

## Figures and Tables

**Fig. 5:**
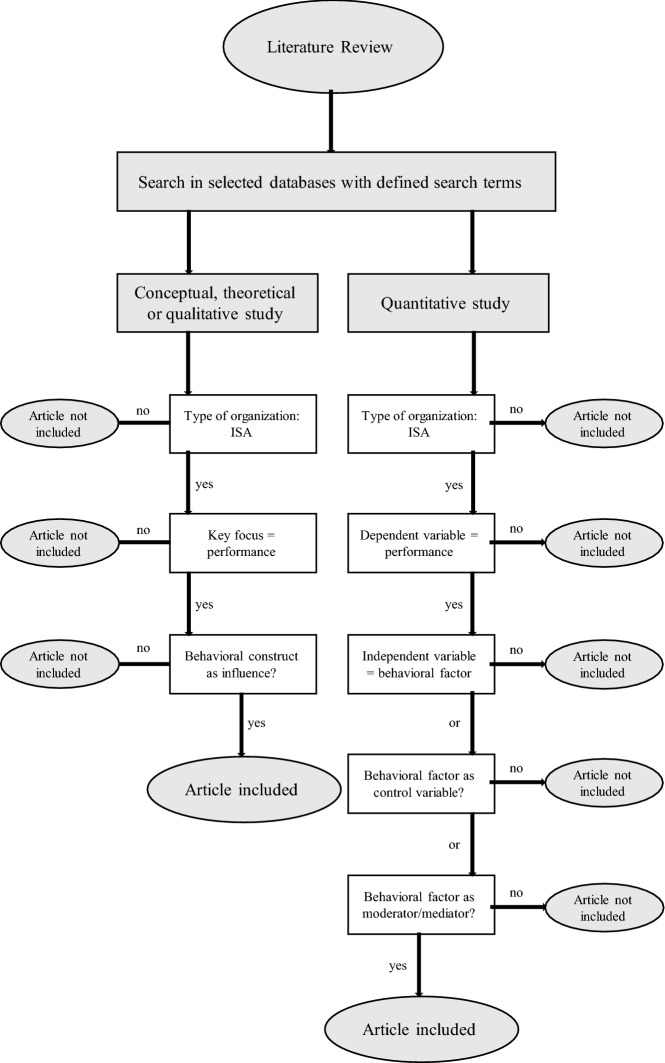
Decision tree

## Appendix

See Fig. 5 and Table

**Table 3:** Bibliographic sources of articles

Journal name	Final selected articles	Impact factor
International Business Review	17	4.270
Journal of International Business Studies	12	7.420
Journal of World Business	5	5.194
Industrial Marketing Management	4	6.510
Journal of Business Research	4	4.874
Journal of International Management	4	3.570
Journal of International Marketing	4	4.200
Organization Science	4	3.860
Strategic Management Journal	4	6.670
The International Journal of	4	3.420
Human Resource Management		
Asia Pacific Business Review	3	0.363
Asia Pacific Journal of Management	3	3.064
International Marketing Review	3	4.610
Journal of Management Studies	3	7.160
Long Range Planning	3	4.041
Academy of Management Perspectives	2	4.160
European Management Journal	2	2.369
Journal of Asia Business Studies	2	1.590
Journal of Construction Engineering and Management	2	2.734
Journal of Transnational Management Development	2	0.740
Research in International Business and Finance	2	1.801
African Journal of Business Management	1	0.570
Asia Pacific Journal of Marketing and Logistics	1	1.680
Aктyaльнi пpoблeми eкoнoмiки	1	0.000
Baltic Journal of Management	1	1.880
Canadian Journal of Administrative Sciences	1	0.940
Chinese Management Studies	1	1.110
Competitiveness Review: An International	1	2.470
Business Journal		
Ekonomika a Management	1	1.210
Engineering, Construction and	1	2.050
Architectural Management		
European Business Review	1	2.340
IBA Business Review	1	0.000
International Entrepreneurship and Management Journal	1	3.670
International Journal of Business and Globalisation	1	0.600
International Journal of Business Performance Management	1	0.540
International Journal of Commerce and Management	1	3.015
International Journal of Conflict Management	1	1.360
International Journal of Construction Management	1	2.250
International Journal of Organizational Analysis	1	1.180
International Journal of Project Management	1	6.620
International Journal of Technology Intelligence and Planning	1	0.530
Journal for Global Business Advancement	1	0.340
Journal of Applied Psychology	1	5.851
Journal of Business & Industrial Marketing	1	2.360
Journal of Euromarketing	1	0.000
Journal of Knowledge Management	1	6.180
Journal of Legal Affairs and Dispute	1	0.000
Resolution in Engineering and Construction		
Journal of Management	1	8.080
Journal of Marketing	1	9.430
Journal of Marketing Channels	1	0.940
Journal of Relationship Marketing	1	0.810
Journal of Strategy and Management	1	1.432
Knowledge Management Research & Practice	1	2.220
Management International Review	1	2.015
Organization Studies	1	3.941
Personnel Review	1	1.710
Project Management Journal	1	2.690
Public Management Review	1	3.970
Studies in Higher Education	1	3.240
The Service Industries Journal	1	1.920
Total Quality Management & Business Excellence	1	2.660
*Total*	*129*	*3.863 (average)*

3.

## Declarations

### Availability of data and material

All relevant datasets generated or analyzed during this study are included in this published article and its supplementary information files. Further datasets generated or analyzed during this study are available from the corresponding author on reasonable request.
